# Identification, Characterization and Immunogenicity of an O-Antigen Capsular Polysaccharide of *Francisella tularensis*

**DOI:** 10.1371/journal.pone.0011060

**Published:** 2010-07-06

**Authors:** Michael A. Apicella, Deborah M. B. Post, Andrew C. Fowler, Bradley D. Jones, Jed A. Rasmussen, Jason R. Hunt, Sayaka Imagawa, Biswa Choudhury, Thomas J. Inzana, Tamara M. Maier, Dara W. Frank, Thomas C. Zahrt, Kathryn Chaloner, Michael P. Jennings, Molly K. McLendon, Bradford W. Gibson

**Affiliations:** 1 Department of Microbiology, University of Iowa, Iowa City, Iowa, United States of America; 2 Buck Institute for Age Research, Novato, California, United States of America; 3 Glycotechnology Core Resource, University of California San Diego, San Diego, California, United States of America; 4 Virginia-Maryland Regional College of Veterinary Medicine, Virginia Polytechnic Institute and State University, Blacksburg, Virginia, United States of America; 5 Center for Biopreparedness and Infectious Diseases, Medical College of Wisconsin, Milwaukee, Wisconsin, United States of America; 6 Department of Biostatistics, College of Public Health, The University of Iowa, Iowa City, Iowa, United States of America; 7 Institute for Glycomics, Griffith University, Gold Coast, Australia; National Institute of Allergy and Infectious Diseases, National Institutes of Health, United States of America

## Abstract

Capsular polysaccharides are important factors in bacterial pathogenesis and have been the target of a number of successful vaccines. *Francisella tularensis* has been considered to express a capsular antigen but none has been isolated or characterized. We have developed a monoclonal antibody, 11B7, which recognizes the capsular polysaccharide of *F. tularensis* migrating on Western blot as a diffuse band between 100 kDa and 250 kDa. The capsule stains poorly on SDS-PAGE with silver stain but can be visualized using ProQ Emerald glycoprotein stain. The capsule appears to be highly conserved among strains of *F. tularensis* as antibody 11B7 bound to the capsule of 14 of 14 *F. tularensis* type A and B strains on Western blot. The capsular material can be isolated essentially free of LPS, is phenol and proteinase K resistant, ethanol precipitable and does not dissociate in sodium dodecyl sulfate. Immunoelectron microscopy with colloidal gold demonstrates 11B7 circumferentially staining the surface of *F. tularensis* which is typical of a polysaccharide capsule. Mass spectrometry, compositional analysis and NMR indicate that the capsule is composed of a polymer of the tetrasaccharide repeat, 4)-α-D-GalNAcAN-(1->4)-α-D-GalNAcAN-(1->3)-β-D-QuiNAc-(1->2)-β-D-Qui4NFm-(1-, which is identical to the previously described *F. tularensis* O-antigen subunit. This indicates that the *F. tularensis* capsule can be classified as an O-antigen capsular polysaccharide. Our studies indicate that *F. tularensis* O-antigen glycosyltransferase mutants do not make a capsule. An *F. tularensis* acyltransferase and an O-antigen polymerase mutant had no evidence of an O-antigen but expressed a capsular antigen. Passive immunization of BALB/c mice with 75 µg of 11B7 protected against a 150 fold lethal challenge of *F. tularensis* LVS. Active immunization of BALB/c mice with 10 µg of capsule showed a similar level of protection. These studies demonstrate that *F. tularensis* produces an O-antigen capsule that may be the basis of a future vaccine.

## Introduction

*Francisella tularensis* is a gram-negative, aerobic, facultative intracellular bacterium and is the etiological agent of tularemia. The organism was first described by McCoy and Corbin in 1911 in Tulare County California [1]. *F. tularensis* is found throughout the Northern hemisphere. Infection with *F. tularensis* can occur by inhalation, insect bite, subcutaneous inoculation through a break in the skin, ingestion of contaminated meat or water, or by animal bite [2]. *F. tularensis* is one of the most infectious bacterial organisms known and as few as 10 organisms can cause disease in humans by inoculation or inhalation [3], [4]. In the United States, the majority of endemic disease occurs in hunters, laboratory personnel and children in rural areas. The highest incidence of disease has occurred in the USA over the past decade in Missouri and Arkansas [5]. Children from age three to ten and adults over 50 have the greatest incidences of disease [5]. Several forms of the disease can occur that depend on the route of infection, dose of bacteria and virulence of the infecting organism, including: ulceroglandular, glandular, oculoglandular, oropharyngeal and pneumonic. Infection with *F. tularensis* is marked with abrupt onset of symptoms, including fever, headache and body aches [2]. Left untreated, infection is associated with high morbidity and mortality.

*F. tularensis* is classified as a Category A biological agent by the Strategic Planning Work Group of the Centers for Disease Control and Prevention as most likely to pose a potential national security risk [5], [6]. *F. tularensis* has the potential to be an efficient agent of biological warfare because it is highly infectious [6]. In addition, the bacterium is stable over a variety of environmental conditions, is easily dispersed as an aerosol, large quantities of the bacterium can be easily manufactured, the general population is susceptible to infection and infections lead to high morbidity and mortality [7]. It is estimated that intentional airborne release of *F. tularensis* into a metropolitan area of a major city would result in major morbidity, mortality and financial loss [7].

Previously, a live attenuated *F. tularensis* vaccine was available to at-risk personnel; however, it did not provide complete protection against all forms of the disease. Vaccinated human volunteers were protected during aerosol infection from the most dangerous typhoidal form of infection, but the incidence of the ulceroglandular form of the disease was not affected; instead, vaccination lessened the severity of the infection [8]. Development of a new vaccine is necessary because of limitations with the current vaccine. These include difficulty in standardizing the vaccine because it is administered via scarification and phenotypic instability of the vaccine strain. Little is known about the necessary protective antigens or what arm of the immune response should be targeted with the vaccine. Recent reports indicate that the vaccine strain may be able to reacquire some virulence characteristics on passage [9]. Therefore, an intensified search is underway to develop a defined subunit vaccine comprised of *Francisella* cell surface components such as protein antigens, lipopolysaccharide (LPS) [10] and/or capsule, or a live vaccine with specific genetic modifications which preclude reversion to virulence.

Capsular antigens have been proven to be the basis for effective vaccines for Gram-negative and Gram-positive human pathogens [11], [12]. *F. tularensis* has been considered to be encapsulated for over 40 years based on smooth to rough colony phenotype transition and evidence of the presence of conserved polysaccharide structures in the organism [13]. Currently, little is known about the chemical structure or antigenic nature of the capsular antigen of *F. tularensis.* A purified capsular preparation has never been described and most published reports dealing with capsule depended on identifying “rough” colonies which were considered unencapsulated [14], [15].

Using a crude “capsular” extract from *F. tularensis* subsp. *tularensis* SCHU S4 as prepared by the method of Hood as an immunogen [16], we developed a bank of monoclonal antibodies and evaluated each for binding to antigens with the characteristics of a typical polysaccharide capsule that help distinguish it from the LPS. The characteristics include being protease and phenol resistant, ethanol precipitable and having a high molecular weight (>100 kDa). Two of these monoclonal antibodies bound a high mass capsule-like structure on Western blot that matched these characteristics. Using one of these antibodies as a probe, we were able to purify this capsular material, free of other bacterial components including LPS. Physiochemical analysis of the antigen indicated that it contains a tetrasaccharide repeat, 2-acetamido-2,6-dideoxy-O-D-glucose (O-QuiNAc), 4,6-dideoxy-4-formamido-D-glucose (O-Qui4NFm), and 2-acetamido-2-deoxy-O-D-galacturonamide (O-GalNAcAN) of similar, if not identical, structure and composition to the LPS O-antigen. Compositional analysis and mass spectrometry studies did not identify Kdo or lipid A as a component of the capsular antigen. Passive immunization with this monoclonal antibody and active immunization of BALB/c mice with the capsule was protective against a challenge of 150-fold the LD_50_ of *F. tularensis* LVS. Immunization of these mice with the capsule resulted in generation of antibodies to the capsule preparation and not the O-antigen subunits of the LPS demonstrating a unique epitopic structure to the capsule. These results indicate that the *F. tularensis* capsule produces an O-antigen capsule which may have the potential to be a protective immunogen against *F. tularensis* infection.

## Results

### Development and characterization of the *F. tularensis* anti-capsular monoclonal antibody

**Figure 1:**
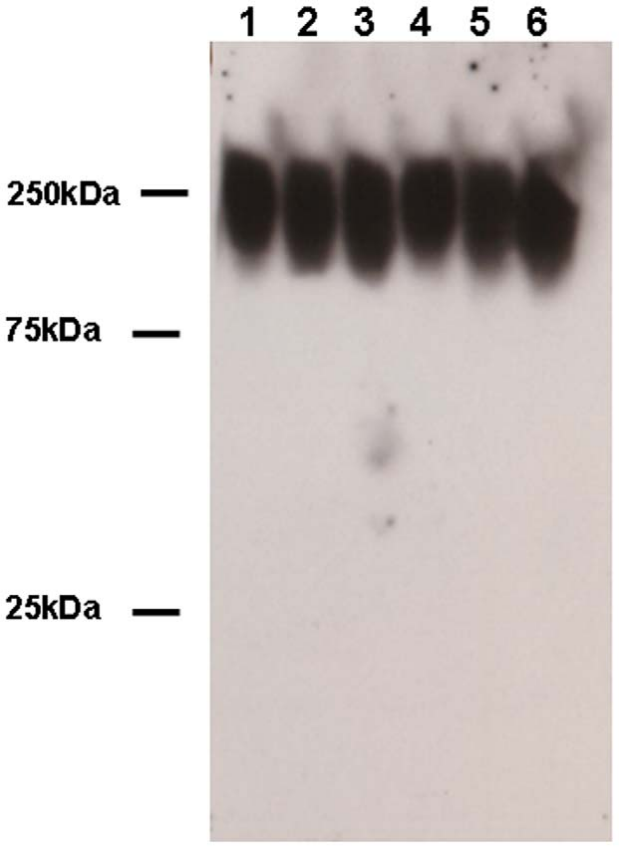
Figure 1 shows a Western blot of six *F. tularensis* strains using MAb 11B7. This antibody binds to capsule from lysates of five *F. tularensis* strains from the Oregon State Health lab (lanes 1-5) and *F. tularensis* subsp *holarctica* 1547 (lane 6).

**Figure 2:**
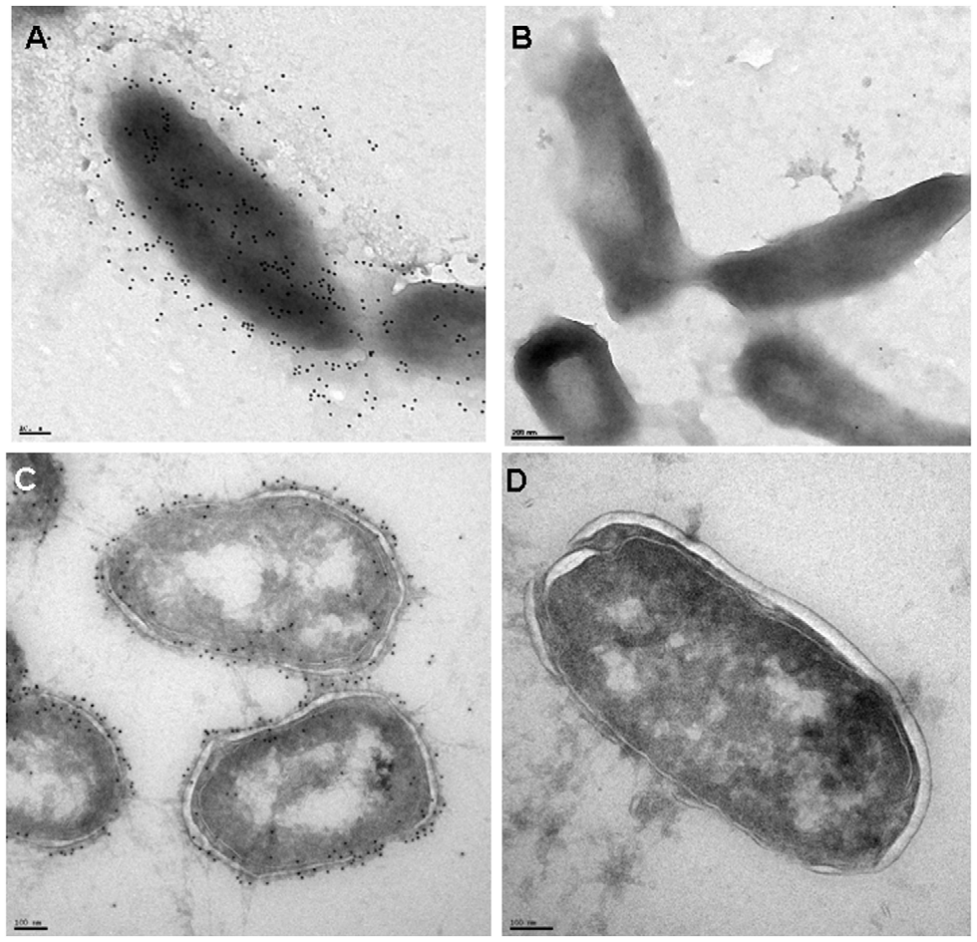
Figure 2 shows the results of immunoelectron microscopic studies of surface labeling with MAb 11B7. Figure 2A is a whole mount immunoelectron microscopy study that demonstrates surface labeling of *F. tularensis* SCHU S4 with 12 nm colloidal gold particles and Figure 2B is a secondary only control for the whole mount study. Figure 2C shows a cryo-immunoelectron micrograph of *F. tularensis* LVS stained with MAb 11B7 and 12 nm colloidal gold. It shows circumferential staining of capsule material on the surface of the organism. Figure 2D shows the secondary antibody control section. Magnification is 15, 000×.

In order to determine if *F. tularensis* SCHU S4 produced a capsular antigen and to develop a probe for it, we elected to make monoclonal antibodies (MAb) to a high salt extract of *F. tularensis* subsp. *tularensis* SCHU S4 prepared according to the method of Hood [16]. This resulted in the identification of antibody 11B7 which recognized a structure with the characteristics of a carbohydrate capsular antigen, i.e., high mass, diffusely migrating, and resistant to proteolytic enzymes and phenol. As can be seen in Figure 1, Western blot analysis indicated that MAb 11B7 bound to a structure between 100 kDa and 250 kDa. Subsequent studies demonstrated that this structure had the characteristics of a polysaccharide capsule. Figure 1 also demonstrates the conserved nature of the putative capsule as five distinct strains of *F. tularensis* obtained as glutaraldehyde-fixed organisms and *F. tularensis* subsp. *holarctica* 1547 reacted to this antibody. Our studies demonstrated that the capsular material was firmly associated with the intact bacteria as only minimal amounts of capsule could be detected in broth culture supernates cleared of organisms at 4, 8 and 24 hours (Figure S1). Subsequent studies indicated that a similar structure could be identified in 15 *F. tularensis* type A and type B strains from a wide geographic distribution in North America (Table 1). We performed immunoelectron microscopy using “whole mount” samples and cryo-immunoelectron microscopy on strains *F. tularensis* SCHU S4, 1547 and LVS using MAb 11B7. A representative stained images of each is shown in Figure 2A and 2C. This demonstrates the presence of circumferential labeling of the organism by this antibody confirming the surface location of the antigen. The secondary antibody controls for both studies show no labeling (Figure 2B and 2D).

**Table 1:** Reactivity of *F. tularensis* strains and mutants with 11B7 and anti-LPS MAb FB11.

Strain	Capsule^1^ positive	LPS^2^ O-antigen
*F. tularensis* SHU S4	Yes	Yes
*F. tularensis* LVS-FDA	Yes	Yes
*F. tularensis* LVS-VT	Yes	Yes
*F. tularensis* 1547	Yes	Yes
*F. tularensis* 1547*msbB*	Yes	No
*F. tularensis* 1547*msbB* [*pmsbB*]	Yes	Yes
*F. tularensis 0673-0674*	Yes	No
*F. tularensis* 1623	Yes	Yes
*F. tularensis* T1 0902	Yes	Yes
*F. tularensis* WY96-3418	Yes	Yes
*F. tularensis* MA00-2987	Yes	Yes
*F. tularensis* NR-50(NIH B-38)	Yes	Yes
*F. tularensis* OSPHL 2001-1011	Yes	Yes
*F. tularensis* OSPHL 2001-1-0513	Yes	Yes
*F. tularensis* OSPHL 2002-1-0990	Yes	Yes
*F. tularensis* OSPHL 2001-1-0074	Yes	Yes
*F. tularensis* OSPHL 2001-1-0143	Yes	Yes
*F. tularensis* LVS*capB*	Yes	Yes
*F. tularensis* LVS*capC*	Yes	Yes
*F. tularensis* LVS*wbtC*	No	No
*F. tularensis* LVS*wbtK*	Yes	Modified*
*F. tularensis* LVS*0708*	No	Yes
*F. tularensis* LVS*wbtM*	No	No
*F. tularensis* LVS*wbtK*	No	No
*F. tularensis* LVS*wbtA1*	No	No
*F. tularensis* LVS*wbtA2*	No	No
*F. tularensis* LVS*wbtI*	No	No
*F. tularensis* LVS *FTT0706*	Yes	No
*F. tularensis* SCHU S4 *0673-0674*	Yes	No
*F. novicida* U112	No	No

*high mass O-antigens absent compared to parent strain.

1 - Reacted with MAb 11B7 to high mass structure on Western blot.

2 - Reacted with MAb FB11 demonstrating O-antigen subunits.

### Characterization of the capsule from *Francisella tularensi*s

**Figure 3:**
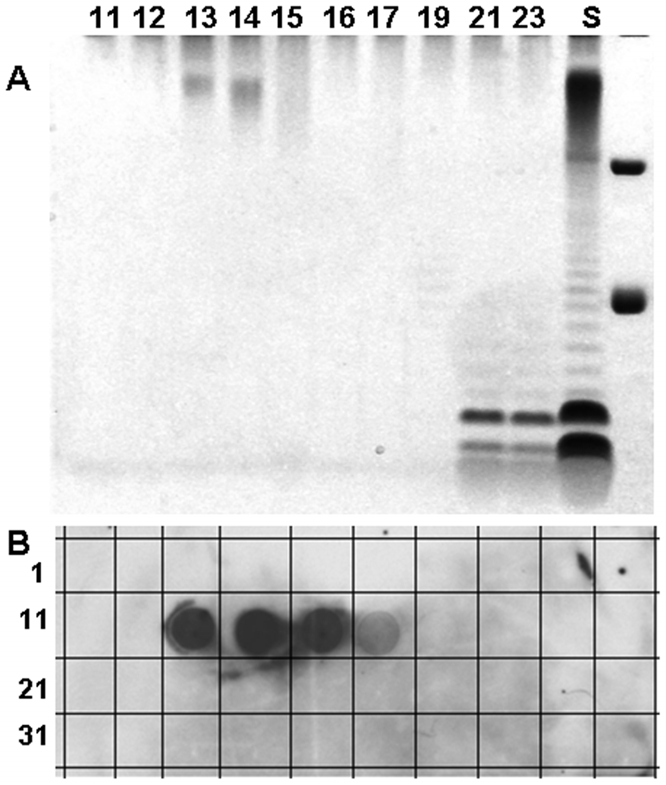
Figure 3 demonstrates chromatographic separation using Sephacryl SH500 that shows the separation of *Francisella* capsule from the LPS. Panel A is a ProQ Emerald green stained SDS-PAGE showing the results of column separation. The capsule can be seen in lanes 13 and 14 and the LPS in lanes 21 and 23. Lane S is an aliquot of the sample after proteinase K and phenol extraction and prior to Triton X-114 extraction. Panel B is an immunodot assay of each fraction using MAb 11B7. The numbers in the panels correspond to aliquots from each one ml fraction from the column.

**Figure 4:**
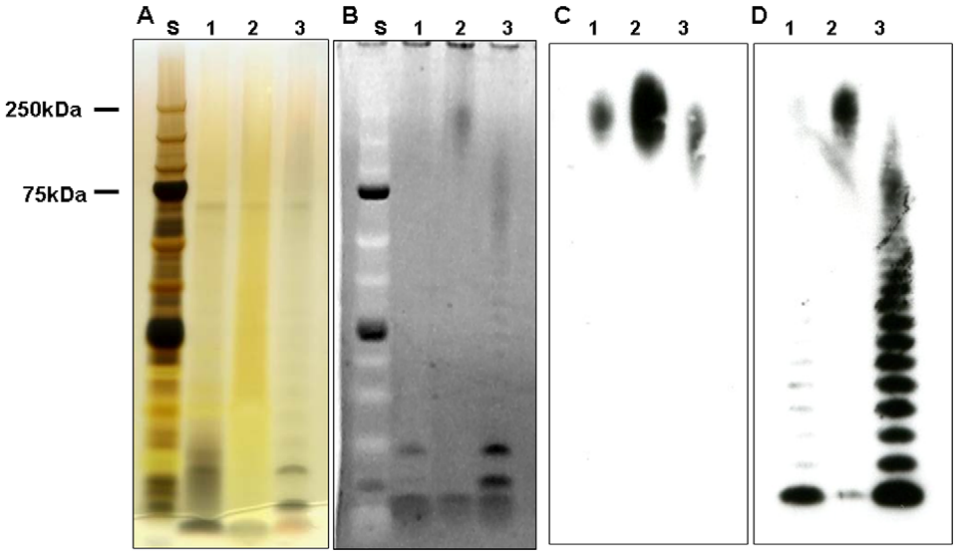
Figure 4 shows a silver stained gel (panel A), ProQ Emerald Green glycostained gel (panel B), a Western blot with the anti-capsule MAb 11B7 (panel C) and a Western blot with MAb anti-LPS MAb FB11 (panel D), Lane S is the molecular weight standard, lane 1 contains the crude capsule prep before Triton X114 treatment, lane 2 contains the capsule preparation after Triton X-114 treatment and lane 3 contains LPS separated into the Triton X114 fraction. This contains minimal amounts of residual capsule.

Using the NaCl extraction method described by Hood [16], followed by proteinase digestion, phenol extraction, Triton X-114 treatment and Sephacryl S500 chromatography, we have been able to isolate the capsule away from contaminating LPS. Figure 3A shows a ProQ Emerald glycostain of fractions from a Sephacryl S-500HR chromatography of a *F. tularensis* SCHU S4 crude capsule prep before treatment with Triton X114. The buffer used contained 100 mM NaCl, 10 mM EDTA, 10 mM Tris, 2% SDS pH 7.4 and the chromatography was performed at 37°C. The column void volume was 11 ml (tube 11) and the bed volume was 30 ml. The capsule eluted between 12 and 16 ml and the LPS eluted at 21 ml. Figure 3B shows an immunodot assay of each fraction using MAb 11B7. The MAb 11B7 reactivity to capsule occurs in tubes 12 through 15. Using MAb 11B7 to track the capsule, we have been able to isolate highly purified capsule in milligram quantities from *F. tularensis* SCHU S4, *F. tularensis* LVS and *F. tularensis* subsp. *holarctica* strain 1547. Figure 4 shows comparative staining of the capsule and LPS with silver stain, ProQ Emerald glycostain, MAb 11B7 and the anti-LPS MAb FB11. As can be seen, the silver stain does not readily show the capsule which is visible in the ProQ Emerald stain. Figure 4 Panel C shows that MAb 11B7 reacts most intensely with lane 2, indicating that this is where the majority of the capsule is found. Small amounts of low mass residual capsule are detected in the LPS preparation taken from the Triton X114 fraction (Figure 4, Panel C, lane 3). The anti-LPS MAb FB11 reacts with both the high molecular weight capsule, and also the O-antigen of the LPS (Figure 4, Panel D, lanes 2 and 3). The absence of a high molecular weight band reacting with this MAb in the LPS lane indicates that there is very little capsule material present in the LPS sample. The absence of the bands corresponding to the O-antigen repeating unit of the LPS in the capsule sample indicates that LPS is essentially absent from the final capsular preparation (Figure 4, Panel D, lane 2).

### Physicochemical analysis of the *F. tularensis* capsule

#### Compositional analyses of the capsule by MALDI-MS and GC-MS

**Figure 5:**
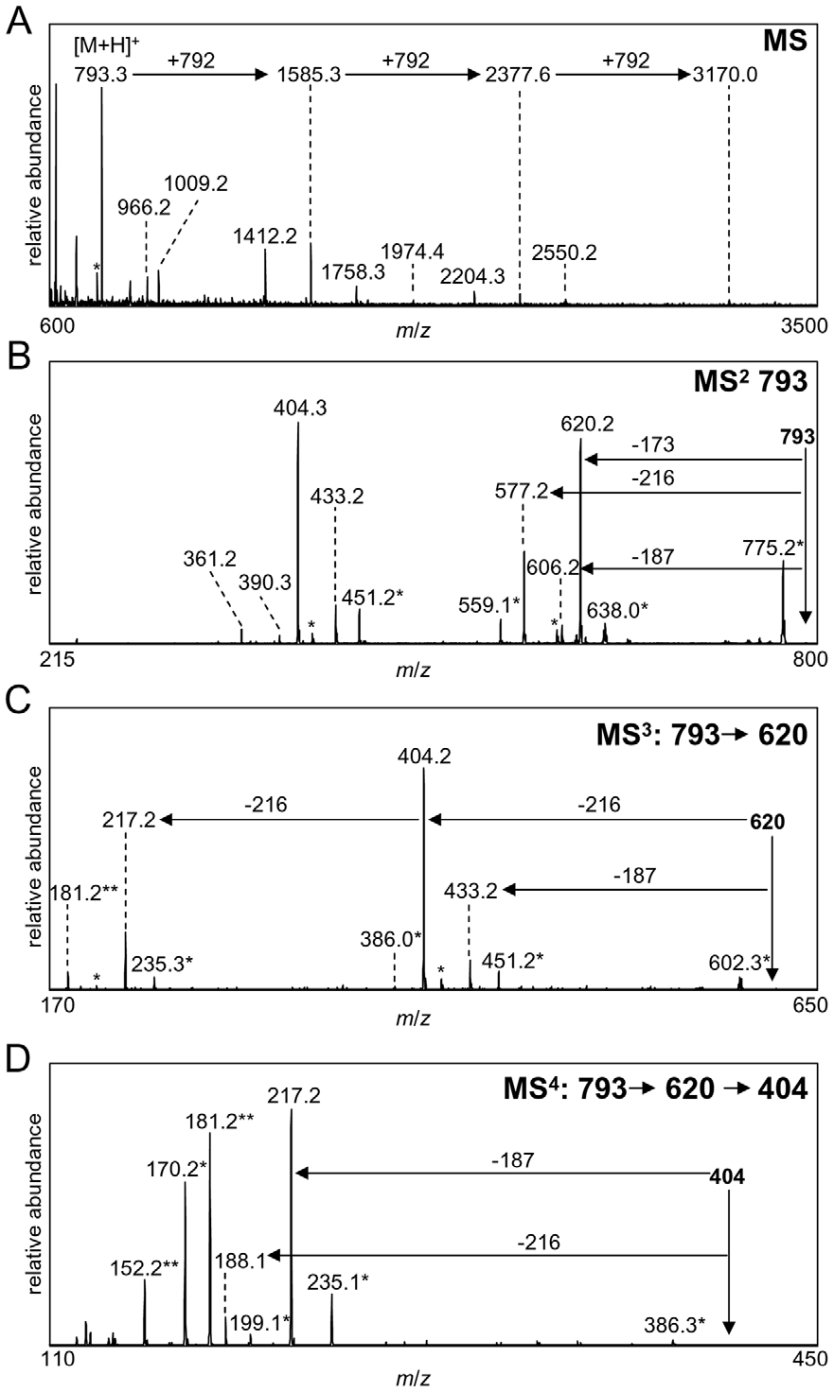
Figure 5 shows positive ion vMALDI-LIT mass spectra of unprocessed capsule. Gas-phase fragmentation of the intact capsule allowed fragments of the capsule to be observed by MS analyses. The MS^n^ spectrum (A) of the capsule demonstrated that the predominant mass observed in the spectrum was at *m/z* 793. The presence of structures consisting of a 792 Da saccharide repeating unit with one (*m/z* 793), two (*m/z* 1585), three (*m/z* 2377), or four (*m/z* 3170) saccharide repeats were detected. MS^n^ analyses, using collisionally induced dissociation, of a single unit tetrasaccharide at *793* m/z (B) yielded a number of fragment ions. The predominant fragment ions were sequentially fragmented to yield MS^3^ data of *m/z* 620 (C) and MS^4^ data of *m/z* 404 (D). The MS^4^ data of *m/z* 404 demonstrated that it is composed of two carbohydrate monomers with nominal masses of 187 and 216 Da, and that these sugars are located adjacent to one another. Masses labeled with an * designate major masses +/− water.

The mass spectrometry (MS) approach we used to provide critical composition and sequence information on the capsule was based on methods we employed earlier for LPS and endotoxin [17], [18]. We examined the capsular material both directly and after limited HF treatment using matrix-assisted laser desorption ionization mass spectrometry (MALDI-MS) on an LTQ linear ion trap instrument coupled to an intermediate vacuum vMALDI ion source. In both cases, initial positive-ion MS data indicated that there was a 792 Dalton (Da) repeat unit. Figure 5 shows MALDI-MS analyses of unprocessed capsule in the positive ion mode. These data show that the predominant protonated species observed in the MS spectrum is one Da higher at *m/z* 793. In addition, monoisotopic masses observed at *m/z* 1585, 2377 and 3170, correspond to the addition of one, two or three of the 792 Da repeating units to the original structure, respectively. Data observed using MALDI-time of flight (MALDI-TOF) analyses, which allowed us to observe a larger mass range than the vMALDI, yielded similar results; a 792 Da repeating unit was observed, with data obtained for up to six repeating units (Figure S2, Table S1). Similar to what was observed for the unprocessed capsule sample, MALDI-MS analyses of the limited HF-treated capsule also showed the presence of a 792 Da repeating unit (Figure S3). In limited HF treatment the capsule underwent chemical hydrolysis to generate oligosaccharide fragments, so unlike the fragments generated in MALDI-MS in the unprocessed capsule which were derived from gas-phase fragmentation, the HF-treated capsule produced peaks shifted up in mass by the addition of water (M = 792+18 = 810).

Multi-stage mass spectrometry (MS^n^) of the unprocessed capsule was utilized to determine the individual components of the repeating unit (Figure 5). MS/MS analyses of the monoisotopic mass [M+H]^+^  = 793 generated two major fragments at *m/z* 620 and 404 and several minor fragments at *m/z* 606, 577, 433, 390, and 361. The mass at *m/z* 620 corresponds to the loss of 173 Da from the *m/z* 793 precursor ion. MS^3^ fragmentation of the *m/z* 620 peak resulted in one major fragment at *m/z* 404 and two minor fragments at *m/z* 433 and 217. The fragment at *m/z* 404 corresponds to the loss of 216 Da from the *m/z* 620 precursor ion. The peak observed at *m/z* 433 corresponds to the loss of 187 Da from the *m/z* 620 precursor ion. MS^4^ fragmentation of the precursor ion at *m/z* 404 showed fragments that corresponded to the loss of either 187 or 216 Da. These data demonstrated that the 792 Da unit is a tetrasaccharide containing two 216 Da components, one 187 Da component, and one 173 Da component. Further analyses using MS^n^ of the various observed fragment ions originating from the protonated tetrasaccharide, [M+H]^+^ = 793, gave several disaccharide fragments that defined various nearest neighbor combinations: 216-216 (*m/z* 433), 187-216 (*m/z* 404), 173-216 (*m/z* 390), and 173-187 (*m/z* 361) (Figure 5, Figures S4 and S5, Table 2). These data demonstrated that the sequence of these constituents was either 187-173-216-216 or 216-216-173-187. This composition and sequence is consistent with the previously published O-antigen structure for this strain of *F. tularensis* [19].

**Table 2:** Proposed compositions of masses observed by MALDI-MS^n^ analyses.

[M+H]^+^_observed_	[M+H]^+^_calculated_	GalNAcAN^1^	QuiNAc^1^	Qui4NFm^1^
793.17	793.32	2	1	1
620.17	620.17	2	1	0
606.17	606.23	2	0	1
577.17	577.24	1	1	1
433.17	433.16	2	0	0
404.25	404.17	1	1	0
390.25	390.15	1	0	1
361.17	361.16	0	1	1

1 GalNAcAN: 2-acetamido-2-deoxy-D-galacturonamide (M = 216.08 Da), QuiNAc: 2-acetamido-2,6-dideoxy-D-glucose (M = 187.09 Da), and Qui4NFm: 4,6-dideoxy-4-formamido-D-glucose (M = 173.07 Da). Masses listed are dehydro forms of each constituent.

Compositional analyses of the capsule monosaccharides by high-pH anion exchange chromatography demonstrated that the capsule sugars were not common monosaccharides, as the major peaks seen in the capsule sample did not correlate with a standard mix of twelve standards (Tables S2 and S3). These data also showed that one of the LPS building blocks, Kdo, was not detected in the capsule sample. Further compositional analyses using GC-MS were also performed. GC-MS analyses of alditol acetate (AA) sugars from the capsule showed the presence of QuiNAc and two additional peaks which likely correspond to anhydro degradation products of Qui4NFm or QuiNAc (6, Panel A). Assignments of the peaks were confirmed by both CI and EI analyses (Figure S6, Panels B-C). Analyses of AA sugars of the LPS gave the same three peaks as the capsule sample, and additional LPS sugars, *N*-acetylglucosamine (GlcNAc) and *N*-acetylgalatosamine (GalNAc) (Figure S7). GC-MS analyses of the capsule as a TMS derivative, confirmed the presence of QuiNAc and HexNAcAN in the sample (Figure S8, Table S4). Supplemental Figure S8 shows the GC-MS analyses of TMS-derived LPS from the same strain of *F. tularensis* from which the capsule was extracted. These data demonstrated that the LPS sample contained glucose, mannose, Kdo, GlcNAc, as well as components of the lipid A (C14:0, C16:0, 3- OH C16:0, and 3-OH C18:0) (Figure S9, Table S5). When these same samples were scanned for amino sugars, the presence of QuiNAc, GalNAc, and GlcNAc was also confirmed. Unlike the LPS sample, none of the LPS-specific components (Kdo or lipid A) were detected in the TMS-derived capsule sample. Partial linkage determination was performed by GC-MS analyses of partially methylated alditol acetates generated from capsule; these data demonstrated the presence of a terminal- and a 3-linked-QuiNAc in this structure (Figure S10).

**Figure 6:**
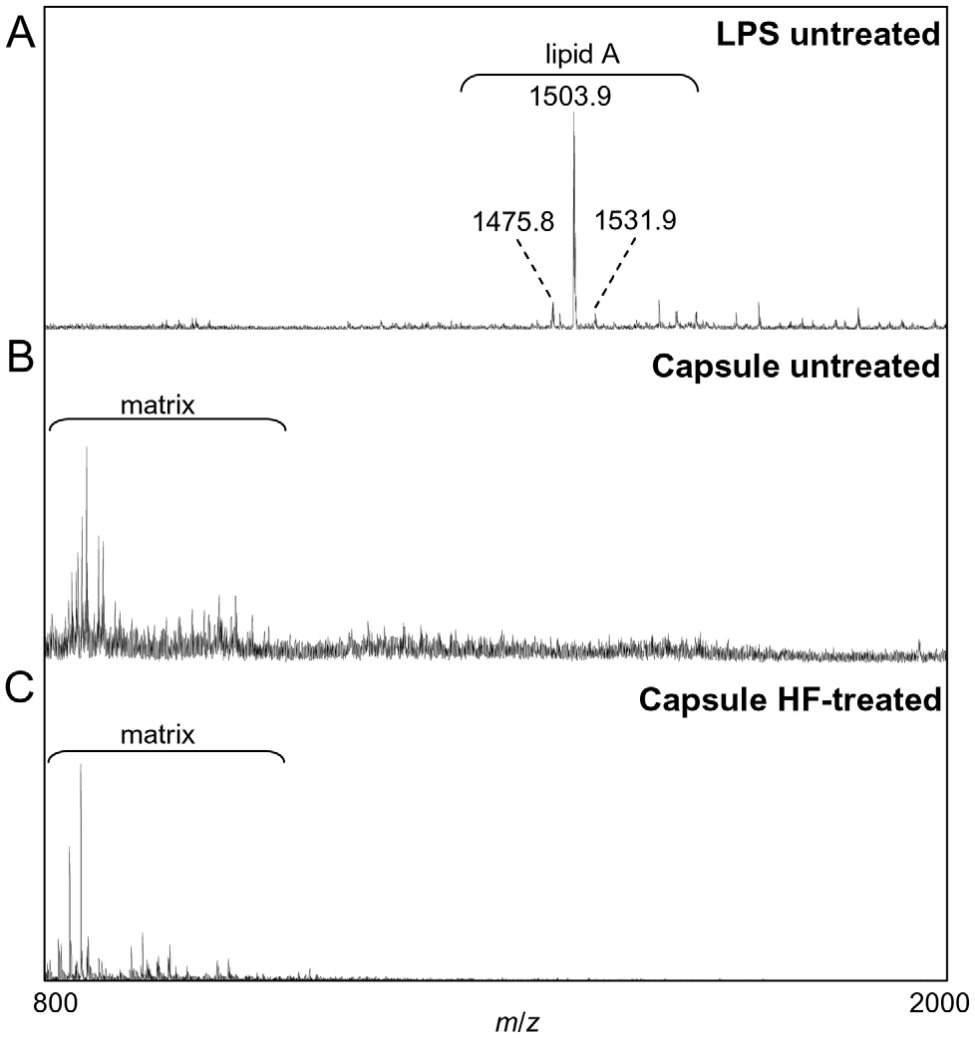
Figure 6 shows a negative-ion vMALDI-MS analyses of LPS (A) and capsule without (B) and with (C) HF treatment. The *F. tularensis* tetraacyl lipid A is readily detected in the LPS sample, but is not seen in either of the capsule samples.

The present studies strongly suggest that the capsule contains the same carbohydrate repeating unit as the O-antigen but that it is a distinct structure from the LPS. Compositional analyses of the LPS and capsule showed that while both the Kdo and lipid A components were clearly present in the LPS sample, they were never detected in the capsule sample. In addition, negative-ion MALDI-MS data of unprocessed LPS and unprocessed and HF-treated capsule showed that the monoisotopic mass corresponding to the previously characterized *F. tularensis* tetraacyl lipid A, [M-H]^-^ = 1504 [18], was readily detected in the LPS sample, whereas no masses corresponding to any species of *F. tularensis* lipid A were detected in any capsule samples (Figure 6). MS^n^ analyses of the LPS sample confirmed that the peak at *m/z* 1504 was lipid A (data not shown). These data coupled with our genetic, chemical, and antibody specificity studies indicate that the capsule is distinct from the *F. tularensis* LPS even though both share the same O-antigen subunit structure.

#### NMR studies

NMR spectra were analyzed to determine if assignments of the capsular extract could be made that are consistent with the carbohydrate composition of the polysaccharide as determined by mass spectrometry. All data are related back to a ^13^C-HMQC experiment. Spin systems were built up starting from the anomeric position and extended sequentially using COSY and short (20 ms) mixing time TOCSY data. A longer (60 ms) mixing time TOCSY data set was used to both extend and further verify sequential connectivities. Connections between spin systems were made using a combination of NOESY data correlating spins near one another in space as well as HMBC to make through bond connections to spins across the glycosidic linkage.

**Figure 7:**
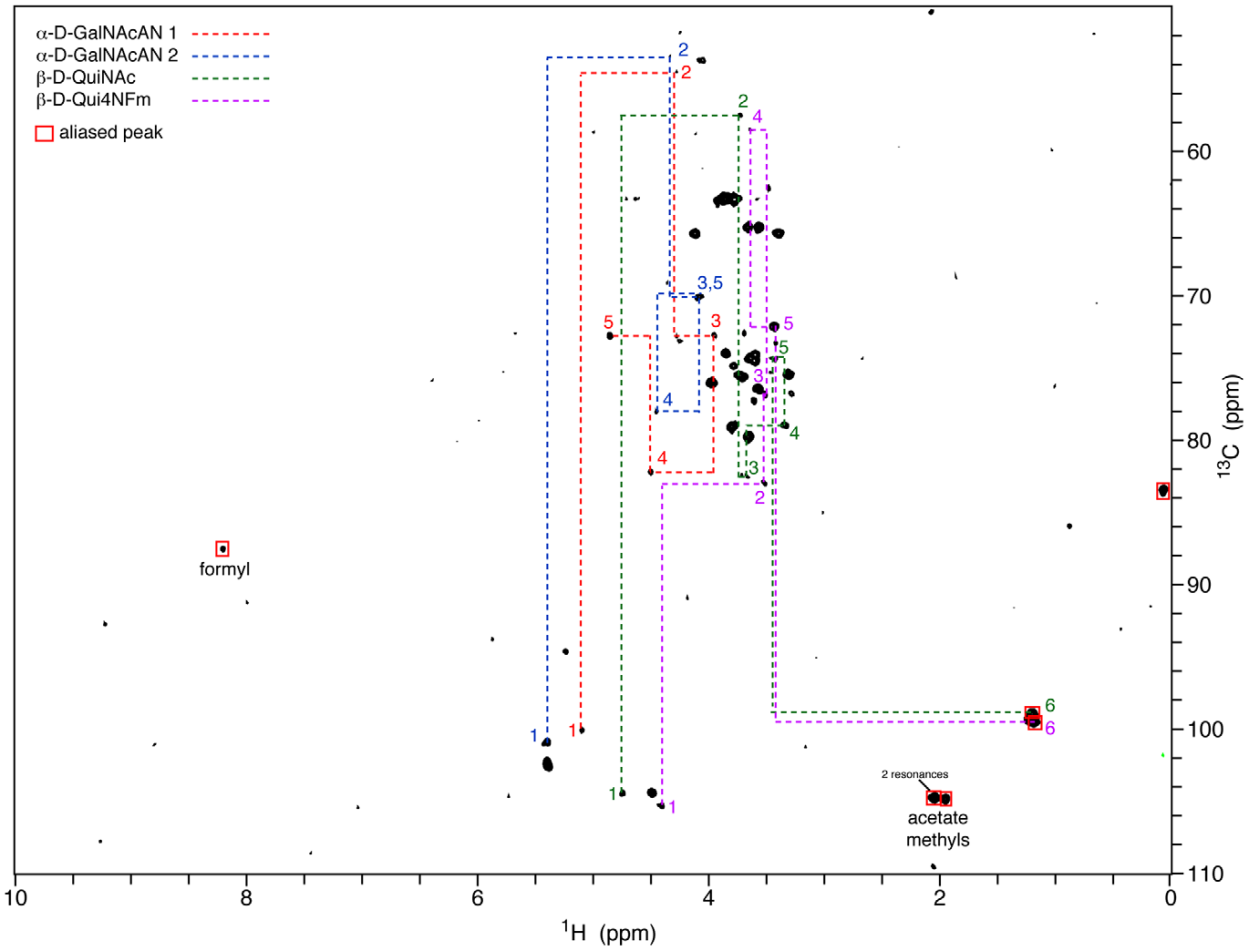
Figure 7 demonstrates ^1^H-^13^C HMQC spectrum of *F. tularensis* capsule polysaccharide collected at 600 MHz and plotted near the noise floor. Methyl and formyl resonances were aliased in the ^13^C dimension to optimize the spectral window. Assignments for each sugar in the core tetrasaccharide are indicated in a different color, and the dashed lines connect the resonances within each spin system. The indicated assignments are highly consistent with those for the O-antigen tetrasaccharide [45], [46].

Analysis of mass spectrometric data indicated a 4 subunit repeat with masses arranged either 216-216-173-187 or 187-173-216-216, as the MS analysis data did not differentiate the reducing and non-reducing ends. There is little data consistent with the first model: no inter-ring HMBC crosspeaks, a single cross-glycosidic NOE of dubious merit, and only three other weak NOEs. However, there is a fair amount of NMR data consistent with the second model, which has a proposed composition 4)-α-D-GalNAcAN-(1->4)-α-D-GalNAcAN-(1->3)-β-D-QuiNAc-(1->2)-β-D-Qui4NFm-(1->. This is the same structure determined by Vinogradov *et al.* for the *F. tularensis* O-antigen [20]. Assignments for the individual spin systems are shown in Table 3 and Figure 7 with the GalNAcAN subunits labeled A and B from the non-reducing to the reducing end of the molecule. Cross-glycosidic HMBC crosspeaks were observed between GalNAcAN(A) C1 and GalNAcAN(B) H4 as well as between GalNAcAN(B) C1 and QuiNAc H3, although no inter-ring HMBC crosspeaks were observed involving Qui4NFm. A number of NOEs that were also observed between subunits are summarized in Table 4.

**Table 3:** NMR Chemical Shift Assignments.

Sugar						
	H1	H2	H3	H4	H5	
α-D-GalNAcAN	*5.09*	*4.07*	*4.26*	*4.51*	*4.86*	
A (non-reducing)	C1	C2	C3	C4	C5	
	*100.1*	*53.7*	*73.1*	*82.2*	*72.8*	
α-D-GalNAcAN	H1	H2	H3	H4	H5	
	*5.4*	*4.24*	*4.02*	*4.44*	*4.04*	
	C1	C2	C3	C4	C5	
	*100.9*	*51.8*	*70*	*78*	*70.1*	
β-D-QuiNAc	H1	H2	H3	H4	H5	H6
	*4.75*	*3.72*	*3.66*	*3.34*	*3.44*	*1.2*
	C1	C2	C3	C4	C5	C6
	*104.4*	*57.5*	*82.4*	*78.9*	*74.4*	*18.9*
β-D-Qui4NFm	H1	H2	H3	H4	H5	H6
	*4.41*	*3.53*	*3.52*	*3.62*	*3.43*	*1.19*
	C1	C2	C3	C4	C5	C6
	*105.3*	*83*	*76.8*	*58.5*	*73.3*	*19.5*

**Table 4:** NOEs Observed Between Sugar Moieties.

Inter-ring NOEs observed (grouped by linkage)		
GalNAcAN(A) H1	GalNAcAN(B) H4	glycosidic
GalNAcAN(B) H1	QuiNAc H3	glycosidic
	QuiNAc H4	
	QuiNAc H6	
GalNAcAN(B) H2	QuiNAc H3	
QuiNAc H1	Qui4NFm H2	glycosidic
Qui4NFm H1	GalNAcAN(A) H4	glycosidic
	GalNAcAN(A) H5	
Qui4NFm H2	GalNAcAN(A) H4	
GalNAcAN(A) H1*	GalNAcAN(B) H3, H5*	
GalNAcAN(A) H2*	GalNAcAN(B) H4*	
QuiNAc H1*	Qui4NFm H1, H3*	
QuiNAcH6*	Qui4NFm H3, H4, and formyl*	
Qui4NFm H1*	GalNAcAN(A) H3*	

Additional possible NOEs*

*the NOEs listed in this section have characteristics that make them less than ideal, *i.e.* they fall as a shoulder of another peak, under an intra-ring NOE, are very weak, or lie near the residual water signal.

One result from the NMR data that was unexpected is the presence of a number of stronger resonances in the HMQC spectrum. The total number of these stronger signals, as well as just those in the anomeric region of the spectrum, would seem to indicate the presence 2 or 3 additional sugars or breakdown products of the identified sugars. The MS data previously described, however, is clean and only indicates the presence of a single tetrasaccharide repeat. A more intense resonance in an NMR spectrum can arise in one of two circumstances. The first is that there is a greater concentration of that spin relative to the rest of the spins in the spectrum. This possible explanation could point to the presence of a contaminant, possibly a short oligosaccharide that co-purified with the capsular polysaccharide. The second way more apparently intense peaks can arise in a spectrum is if the spins giving rise to those resonances are more mobile (*i.e.* faster relaxing) than the rest. This explanation could point to a more flexible substituent branched off the main polysaccharide. Which possible explanation, if either, is correct remains unclear.

#### Studies of capsule expression in *F. tularensis* biosynthesis pathway mutants

**Figure 8:**
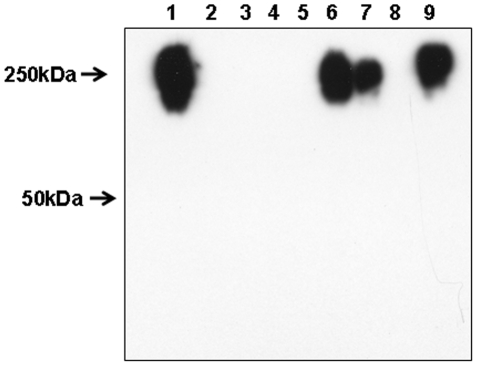
Figure 8 is a Western blot developed with MAb 11B7 showing whole organism lysates from *F. tularensis* 1547 (lane 1), *F. tularensis* LVS *wbtI, wbtA2, wbtA1, capB, capC, and wbtC* in lanes 2–7 respectively. *F. tularensis* LVS is in lane 8.

**Figure 9:**
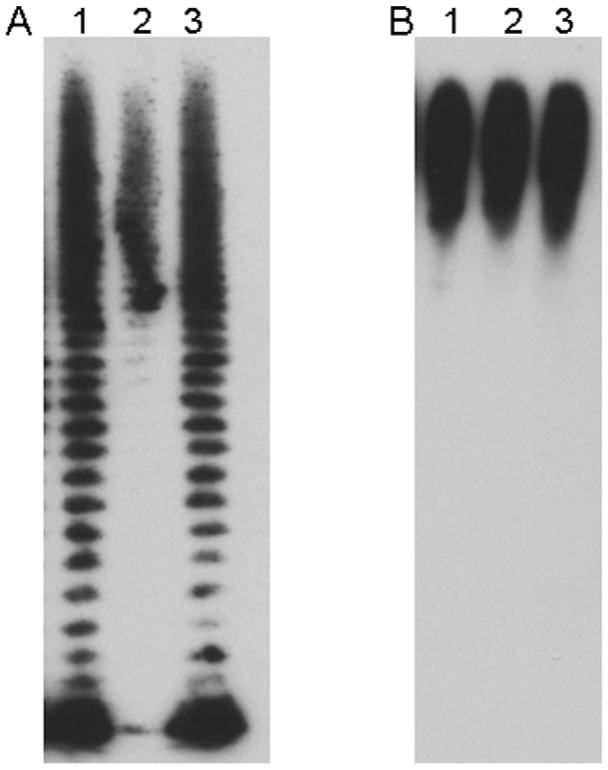
Figure 9 is a Western blot which demonstrates loss of O-antigens but persistence of capsule in *F. tularensis lpxL*. Panels A and B show *F. tularensis* LVS (lane 1), *F. tularensis 1547lpxL (lane 2)* and *F. tularensis 1547lpxL* complemented with *lpxL* (lane 3). Panel A was reacted with the *F. tularensis* anti-LPS MAb FB11 and panel B was reacted with the *F. tularensis* anti-capsule MAb 11B7.

Using mutants in *F. tularensis* LVS of glycosyltransferases and putative capsule biosynthesis genes (*cap*) obtained from Dr. Dara Frank of the Medical College of Wisconsin, we were able to confirm that the LPS O-antigen biosynthesis pathway plays a role in the biosynthesis of the *F. tularensis* capsule [21]. These studies have demonstrated that mutations that disrupt glycosyltransferase genes involved in O-antigen biosynthesis (*wbtI, wbtA1, wbtA2, wbtM, wbtI* and *wbtC*) resulted in the loss of capsule expression (Figure 8). Mutations in *F. tularensis* LVS *capB and capC* had no effect on capsule expression. However, a mutation in *wbtK* resulted in loss of high mass O-antigen expression but did not affect capsule expression (data not shown). The Kdo-dependent acyltransferase mutant, *F. tularensis lpxL*, which expresses a portion of the LPS core region but no O-antigen, still produces capsule (Figure 9). *F. tularensis* LVS *FTL0706,* which has complete homology to the O-antigen polymerase of *F. novicida*, does not express O-antigens yet still expresses the capsule (Table 1). In addition, a Tn*5* mutation in the promoter region of *F. tularensis* SCHU S4 between open reading frames FTT0673-0674 resulted in a mutant that produced a capsule but the mutant does not express a full length O-antigen. FTT0673c has been annotated as a gene of unknown function and FTT0674 is annotated as a ribose-phosphate pyrophosphokinase. These studies demonstrate that O-antigen and capsule are linked at a number of points in the biosynthetic and assembly pathway but there appear to be several points in transport and assembly that are different.

#### Passive immunization of BALB/c mice with 11B7 MAb against challenge with *F. tularensis* LVS

**Figure 10:**
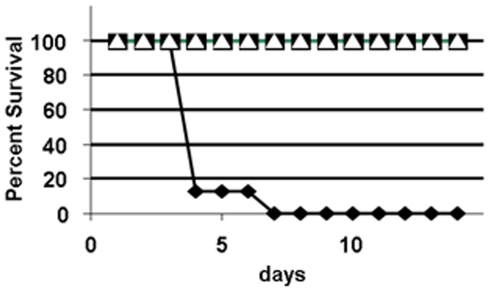
Figure 10 shows the results of protection of BALB/c mice passively immunized with MAb 11B7 from lethal infection due to *F. tularensis* LVS. Five mice received 75 µg of MAb 11B7 and were challenged with 1× 10^4^ cfu (▪), five mice received 75 µg of MAb 11B7 and were challenged with 1× 10^5^ cfu (▵) and five mice received 75 µg of MAb 2C3 a *Neisseria* H.8 MAb as a negative control (⧫) and were challenged with 10^4^ cfu. As can be seen, MAb 11B7 was protective while all of the mice receiving the control antibody died by day 6 after challenge.

We performed an experiment to determine whether mice would be protected from lethal *Francisella* infection by administration of purified monoclonal antibody 11B7. Two groups of five mice received 75 µg of purified MAb 11B7 and 24 hours later were challenged intraperitoneally with either 5×10^4^ or 5×10^5^ colony forming units (cfu) of *F. tularensis* LVS. A third group of mice received 75 µg of a control isotype matched MAb 2C3 that binds to a *Neisseria gonorrhoeae* membrane protein. All animals were followed for two weeks after challenge. As shown in Figure 10, administration of MAb 11B7 provided complete protection to BALB/c mice against >100 LD_50_ doses of *F. tularensis* LVS for more than 14 days, while all of the mice receiving the control MAb 2C3 succumbed to the infection by day 6. In Figure 10, survival of all mice given MAb 11B7 was significantly different from the MAb 2C3 control mice (log-rank p = 5.1×10^−5^), and survival of immunized mice at any one of the two doses of MAb 11B7 was also significantly different from the MAb 2C3 control group (p = 1.6×10^-4^). The passively immunized mice remained active, did not develop ruffled fur or stop eating or drinking.

#### Active immunization of BALB/c mice with capsule and challenge with *F. tularensis*

**Figure 11:**
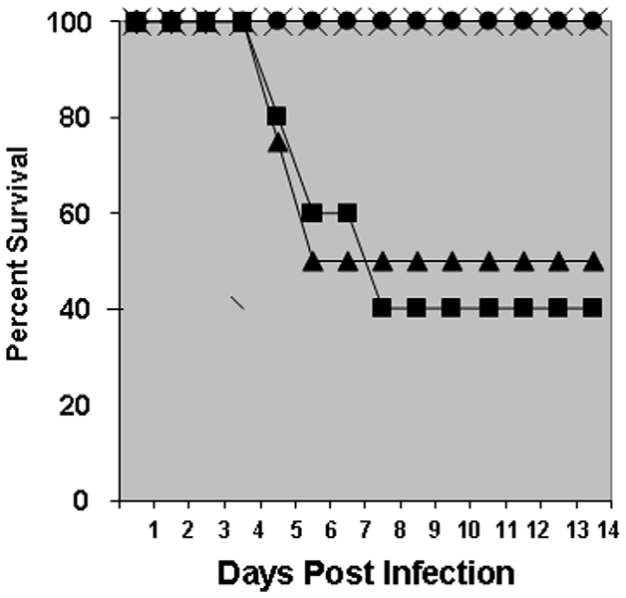
Figure 11 shows the results of immunization of BALB/c mice with 2 doses of 10 µg *F. tularensis* capsular antigen at a four week interval. Capsule immunized mice were challenged a week after the second dose with either 1× 10^4^ (×) or 5× 10^5^ (•) *F. tularensis* LVS. Control mice received PBS and were challenged with either 250 cfu (▴) or 750 cfu (▪) of *F. tularensis* LVS. As can be seen, these results indicate that the capsular antigen is protective against a challenge 666-fold higher than the LD_50_ for *F. tularensis* in mice. Each group contains 5 mice.

To determine if the *F. tularensis* capsule could protect mice against a lethal challenge, BALB/c mice were immunized intraperitoneally twice 30 days apart with 50 µl containing 10 µg of capsule in PBS mixed with an equal amount of TiterMax Gold adjuvant. Control mice received 50 µl of PBS mixed with the same adjuvant on the same schedule. Eight days after the second injection, a group of five of the control mice was challenged with 250 cfu of *F. tularensis* LVS. A second group of four control mice received 750 cfu of *F. tularensis* LVS. One group of five mice immunized with capsule was challenged with 5×10^4^ cfu and a second group of five immunized mice also immunized with capsule received 5×10^5^ cfu of *F. tularensis* LVS. Figure 11 shows the results of these studies. These data demonstrated that in the PBS controls samples, 50% of the control mice receiving 250 cfu and 60% of the control mice receiving 750 cfu, died by day 5. All of the mice immunized with capsule survived a challenge of up to 5×10^5^ *F. tularensis* LVS with no evidence of infection and were euthanized at day 14. In Figure 11, in spite of the fact that the capsule immunized group of mice received a dose of LVS up to 666 times greater than the PBS control animals, survival of all mice immunized with capsule was significantly different from all the control mice (p = 6.9×10^-3^). The actively immunized mice remained active, did not develop ruffled fur or stop eating or drinking.

**Figure 12:**
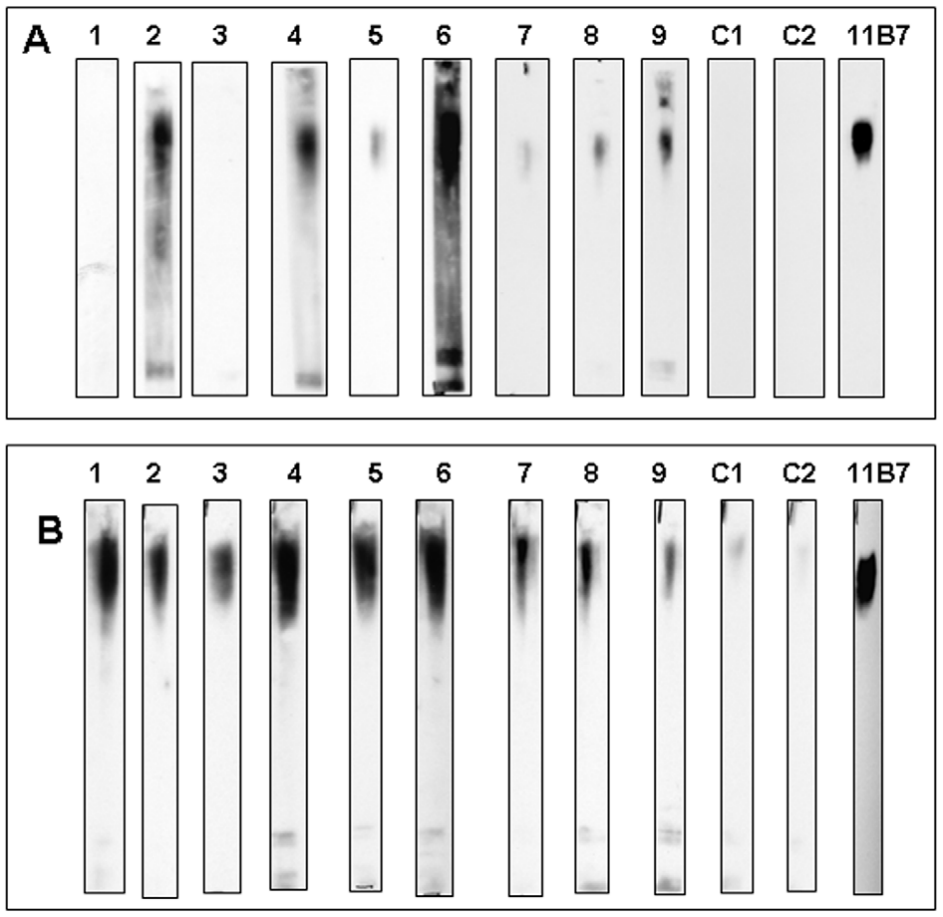
Figure 12 is a composite Western blot using sera from capsule immunized mice. A goat anti-mouse IgG peroxidase conjugate was used to develop panel A and a goat anti-mouse IgM peroxidase conjugate was used in panel B. All sera were tested at a 1∶1000 dilution. C1 and C2 represent sera from animals given PBS only. One strip has been reacted with MAb 11B7 as a control for capsule using the goat anti-mouse IgG peroxidase conjugate.

Western blot analyses (Figure 12) and ELISA performed on sera from the immunized mice demonstrated that all had IgG and IgM reactivity to the capsule and no detectable reactivity to *F. tularensis* LPS. Simultaneous ELISA studies showed that antibody persisted after vaccination for at least 14 days.

## Discussion

Capsular polysaccharides have been shown to be important virulence factors in a wide range of bacterial species. *F. tularensis* has been considered to be encapsulated but the precise nature of the capsular antigen has never been elucidated. Unreferenced comments in Zinsser Microbiology [22], state that there is a single immunotype of *F. tularensis.* Carlisle in 1962 used Ouchterlony plates to compare the immunogenicity of crude extracts from four strains of *F. tularensis* and a “polysaccharide” isolated from a fifth strain [13]. These studies showed immunological identity between the polysaccharide fraction and the four bacterial extracts, leading to the conclusion that there was a common carbohydrate structure in the five *F. tularensis* strains. Hood studied a relatively unrefined extract of *F. tularensis* and did preliminary investigation of sugars isolated from whole bacteria and a “capsular” preparation [16]. His studies found heptose, glucose, galactose, mannose, rhamnose and possibly dideoxy sugars in the cell wall and mannose, rhamnose and possibly dideoxy sugars in the “capsular” preparation. No further characterization was performed. Studies by Sorokin and Sandstrom have shown that acapsular (based on colony morphology and electron microscopy) *F. tularensis* strains are sensitive to killing by bactericidal antibody [14], [15]. Sorokin did not describe how the acapsular status of the strain was produced or verified. Sandstrom used acridine orange mutagenesis to create a mutant library which was screened for "rough" colonies on solid media; the “rough” colonies were designated as lacking capsule. Electron micrographs showed changes consistent with loss of a capsular structure [15] in the mutants that were selected. However, no studies of the composition or the nature of the mutations leading to the phenotype were described. Using tagged mutagenesis, Su and colleagues recently showed that mutations in an operon, they designated *capBCA,* led to an avirulent phenoype in mice [23]. They considered these to be putative capsule genes because of their partial homology to the poly-γ-glutamate capsule biosynthesis locus of *Bacillus anthracis.* Our studies would suggest that this locus plays no role in the synthesis of the *F. tularensis* O-antigen capsular polysaccharide (Figure 8). Whether it encodes for a second capsule-like antigen or another virulence factor is unclear.

We have demonstrated a method for the purification of the *F. tularensis* capsular polysaccharide and have demonstrated that *F. tularensis* type A and type B strains produce an O-antigen capsule composed of repeating O-antigen subunits, 4)-α-D-GalNAcAN-(1->4)-α-D-GalNAcAN-(1->3)-β-D-QuiNAc-(1->2)-β-D-Qui4NFm-(1-. Monoclonal antibody FB11, which reacts with the O-antigen, also binds to the capsule. We believe our success in identifying the capsule was due to developing the MAb 11B7 which permitted us to use it as a means of tracking the polysaccharide during isolation since in addition to the O-antigen epitopic structure, this capsule also has an epitopic structure distinct from the LPS as defined by monoclonal antibody 11B7. This antibody was generated by immunizing mice with the capsule preparation which subsequently resulted in the evolution of antibody 11B7. This antibody appears to react only with the capsule suggesting that this is the immunodominant epitope on that structure. It should be noted that in our monoclonal antibody development, using material obtained from saline washed cells as the immunogen, we did not raise an antibody to the O-antigens, suggesting that the LPS was probably in very low amounts or absent from the preparation.

Our studies have shown that the *F. tularensis* capsule is a repeating polymer of the O-antigen of the LPS. Goldman and co-workers first showed that *Escherichia coli* 0111 expressed a non-LPS surface polysaccharide composed of O-antigens [24], [25]. Subsequently, a number of Gram-negative bacteria have been shown to express similar structures which consist of large repeating subunits not linked to the LPS core region or lipid A [26], [27], [28], [29]. A review by Whitfield expands the description of the different capsule types and segregates them into specific Groups based on their characteristic structures [30]. Group 1 and 4 capsules of *Enterobacteriaceae* are composed of LPS O-antigens [30]. In strains in which the capsule structure is linked to a lipid A core, it is termed a K_LPS_ to distinguish it from the LPS molecules on the same organism. Other strains may have O-antigen capsules that lack the lipid A core but have another yet unidentified structure which anchors them to the cell wall. These are known as K antigens. Our studies failed to detect lipid A or any of the LPS core sugars as a component of the capsule indicating that the *Francisella* capsule is probably a K antigen. *E. coli* group 1 capsules are acidic and typically contain uronic acid structures while group 4 capsules tend to be more diverse and can be distinguished from group 1 capsules by the presence of acetamido sugars in their repeat subunits [30]. The *F. tularensis* capsule fits this characteristic by having two 2-acetamido-2,6-dideoxy-O-D-glucose (O-QuiNAc), 4,6-dideoxy-4-formamido-D-glucose (O-Qui4NFm), and 2-acetamido-2-deoxy-O-D-galacturonamide (O-GalNAcAN) in each O-antigen tetrasaccharide subunit. The genetic organization of capsular genes from a number of bacterial species have been described [31]. The assembly and transport systems involved in production of these capsules are complex and include activators of sugar precursors, glycosyltransferases and then a complex of transport and assembly proteins to move these large macromolecules from the periplasm to the cell surface. Group 4 capsule initiation involves transfer of *N*-acetylglucosamine-1-phosphate to undecaprenol phosphate by WecA followed by the addition of the individual components of the capsular O-antigen subunit to this lipid carrier. The polymerization systems, periplasmic ligase and transport proteins appear to be similar for the group 1 and 4 systems. However, the lengths of the O-antigen repeats in the respective capsules are different. In group 1 the capsule repeats tend to be limited to one or a few O-antigen subunits while the group 4 capsules tend to form long chain O-antigen repeats. The difference in K_LPS_ chain lengths in group 1 and 4 is considered to be due to the absence of *wzz* (the O-antigen chain length determinant) in isolates with group 1 capsules [32]. In an analysis of the *F. tularensis* SCHU S4 genome using the hidden Markov model, we have homologs of proteins involved in assembly of the capsule, WbtB, Wzx, Wzb and WecA, but no proteins in *F. tularensis* with homologies to capsular transport proteins in the enteric family, gammaproteobacteria.

Our observation that the *F. tularensis* lipid A acyltransferase mutant, *lpxL* and *F. tularensis* LVS O-antigen polymerase mutant, *FTL_0706,* did not express repeating O-antigens is not surprising. Analysis of the Western blot using the O-antigen monoclonal antibodies indicated that the polymerase mutant produced an LPS consisting of a core region plus one O-antigen subunit. The fact that the capsule was still expressed in the O-antigen polymerase mutant indicates that the pathways for the polymerization processes are different and suggests that the O-antigen ligase and polymerase are distinct genes in *Francisella*. The *lpxL* mutant expresses only a portion of the LPS core region, but still produces a capsule. The reason for the loss of O-antigen expression in the *lpxL* mutant is less obvious than with the polymerase mutant. Studies in a similar acyltransferase mutant in *Neisseria meningitidis* serogroup B resulted in an organism which failed to transport LPS to the bacterial surface [33]. It is known that encapsulated Gram-negative bacteria can survive without LPS in the outer membrane [34]. In the *N. meningitidis lpxL* mutant, evidence of the LPS terminal structures could be identified accumulating in the cytoplasm of the bacterium while no LPS could be identified on the cell surface. This would suggest that in *F. tularensis lpxL* the O-antigen defect may be related either to inability of the LPS to be transported out of the cell interior or to a failure of the polymerase to add additional O-antigen subunits to the nascent core-O-antigen structure. These results would indicate that the transport pathway and/or assembly pathways for the LPS and capsule are distinct.

Finally, the active and passive immunization studies indicate that the epitope defined by 11B7 is protective against a lethal challenge of *F. tularensis* LVS in mice. The post-immunization sera indicated that the only epitopic structure detectable was to the capsule in spite of the fact that it is composed of O-antigen subunits. Our IgG and IgM studies in the immunized mice suggested that there was evidence of both isotypes of antibody being generated. At this time, it is premature to determine if both or one of the two isotypes provided the protection that was demonstrated.

These studies provide evidence for a capsular polysaccharide of *F. tularensis.* This polysaccharide is a polymer composed of the LPS O-antigen subunit, 2-acetamido-2,6-dideoxy-o-glucose (o-QuiNAc), 4,6-dideoxy-4-formamido-D-glucose (o-Qui4NFm), and 2-acetamido-2-deoxy-o-galacturonamide (o-GalNAcAN). It appears to be conserved across *F. tularensis* type A and B strains. Passive and active protection assays suggest that this may be a useful immunogen to protect against lethal infection. Future studies will include immunization of BALB/c mice with these capsular preparations by the intraperitoneal and intranasal route followed by intranasal challenge with *F. tularensis* SCHU S4 and other more virulent strains to more fully determine the usefulness of these immunogens as a vaccine.

## Materials and Methods

### Strains and culture conditions

Bacterial strains used in this study are listed in Table 5. All *F. tularensis* strains were grown at 37°C on chocolate agar medium supplemented with IsoVitaleX for a final cysteine concentration of 0.1%.

**Table 5:** Strains used in this study.

Strain	Subspecies	source	ref
*F. tularensis* SCHU S4	*tularensis*	BEI	
*F. tularensis* LVS-FDA	*holarctica*	FDA	[47]
*F. tularensis* LVS-VT	*holarctica*	Virginia Tech	[48]
*F. tularensis* 1547	*holarctica*	University of Iowa	[49]
*F. tularensis* 1547*lpxL*	*holarctica*	University of Iowa	[49]
*F. tularensis* 1547*lpxL* [*plpxL*]	*holarctica*	University of Iowa	[49]
*F. tularensis 0673-0674*	*tularensis*	University of Iowa	Apicella
*F. tularensis* 1623	*holarctica*	University of Iowa	
*F. tularensis* T1 0902	*tularensis*	Virginia Tech	
*F. tularensis* WY96-3418	*tularensis*	BEI	
*F. tularensis* MA00-2987	*tularensis*	BEI	
*F. tularensis* NR-50(NIH B-38)	*tularensis*	BEI	
*F. tularensis* OSPHL 2001-1011	*tularensis*	Oregon State Health Lab	
*F. tularensis* OSPHL 2001-1-0513	*tularensis*	Oregon State Health Lab	
*F. tularensis* OSPHL 2002-1-0990	not typed	Oregon State Health Lab	
*F. tularensis* OSPHL 2001-1-0074	not typed	Oregon State Health Lab	
*F. tularensis* OSPHL 2001-1-0143	not typed	Oregon State Health Lab	
*F. tularensis* LVS*capB*	*holarctica*	Medical College of Wisconsin	[50]
*F. tularensis* LVS*capC*	*holarctica*	Medical College of Wisconsin	[50]
*F. tularensis* LVS*wbtC*	*holarctica*	Medical College of Wisconsin	[50]
*F. tularensis* LVS*wbtK*	*holarctica*	Medical College of Wisconsin	[50]
*F. tularensis* LVS*0708*	*holarctica*	Medical College of Wisconsin	[50]
*F. tularensis* LVS*wbtM*	*holarctica*	Medical College of Wisconsin	[50], [51]
*F. tularensis* LVS*wbtK*	*holarctica*	Medical College of Wisconsin	[50]
*F. tularensis* LVS*wbtA1*	*holarctica*	Medical College of Wisconsin	[50]
*F. tularensis* LVS*wbtA2*	*holarctica*	Medical College of Wisconsin	[50]
*F. tularensis* LVS *FTT0706*	*holarctica*	Medical college of Wisconsin	[39]
*F. tularensis* LVS*wbtI*	*holarctica*	Virginia Tech	
*F. tularensis* SCHU S4 *0673-0674*	*tularensis*	University of Iowa	
*F. novicida* U112	*tularensis*	ATCC	

### Transposon selection protocol

A *Francisella*-specific Tn*5* transposon system has been developed by our group that is delivered by a temperature sensitive plasmid [35]. The plasmid carrying the transposon was purified and introduced into *F. tularensis* SCHU S4 by cryotransformation [36]. Colonies obtained after ∼3 days growth at 30°C on MMH agar containing 25 µg/ml spectinomycin were inoculated into 5 ml MMH broth with 25 µg/ml spectinomycin and were grown at 30°C with agitation to an OD_600_ of ∼0.2. Selection of *F. tularensis* SCHU S4 transposition events was performed at 40°C with ∼10^8^ organisms plated. Since transposition frequency has previously been shown to be ∼10^−3^, approximately 10^5^ random chromosomal transposon mutants were created by this procedure. Individual *F. tularensis* Tn*5* mutant colonies (∼7,500 mutants) were picked and arrayed into 96-well cell culture plates in 100 µl MMH broth and were incubated at 37°C until turbid. Freezer stocks were made by adding 100 µl of 2X freezing medium (1.0 M sucrose, 20% glycerol).

### Development of hybridomas producing antibodies to the capsule of *F. tularensis*

BALB/c mice were immunized intraperitoneally with 25 µl of a 1 mg/ml solution of a high salt extract of *F. tularensis* subsp. *tularensis* SCHU S4 prepared according to the method of Hood [16]. Animals were reimmunized at day 21 and day 28. Sera from the immunized mice was tested for antibodies to the immuning extract in ELISA. After confirmation of high antibody titers (>1∶10,000), animals were sacrificed 4 days later and their spleens removed. The splenocytes were recovered and fused to SP2/0 murine myeloma cells using 40% polyethylene glycol (1540 MW). Selection was accomplished by plating the fused cells in the presence of hypoxanthine-aminopterin-thymidine DMEM and plated in limiting dilutions to achieve approximately 1 - 5 cells per well. When colonies appeared, supernatants were tested for presence of antibodies against the saline extract in ELISA. Supernates which proved to be positive in ELISA were tested in Western blot. After this testing, MAb 11B7 fit our criteria as a possible anti-capsular antibody. MAb 11B7 was determined to be an IgG1k using Isostrips (Santa Cruz Biotechology, Inc, Santa Cruz, CA). MAb 11B7 was recloned to assure purity and antibodies produced from these lines for use in this study.

### Screening mutants for reduced capsule and identification of the transposon insertion site

In the BSL-3 facility, *F. tularensis* SCHU S4 Tn*5* mutants were inoculated into duplicate 96 well dishes containing MMH broth and were grown until turbid. Cultures were killed by adding 100 µl of 4% paraformaldehyde to each well and incubating overnight. After ensuring sterility, the plates were removed from the BSL-3 facility and the liquid was removed by evaporation. The capsule produced by each organism was then quantitatively determined by ELISA using capsule-specific antibody.

For *F. tularensis* transposon mutants with reduced capsule production, chromosomal DNA was isolated from individual mutants and digested with *Eco*RI to create a DNA fragment with the *ori*R6K origin, the *aphA3* gene and flanking chromosomal sequence. The digested DNA was ligated, transformed into a *pir* ^+^ *E. coli* strain and plated onto agar plates with kanamycin to select for transformants that carried the plasmid of interest. Plasmid DNA was isolated and sequenced using a primer with the sequence 5′CATGCAAGCTTCAGGGTTGAG 3′ that anneals to the 3′ end of the *aphA3* gene and produced sequence of the flanking chromosomal DNA. Sequence data was used to search the sequenced bacterial chromosomal database using NCBI BLAST to identify Tn*5* insertion sites within the *F. tularensis* chromosome.

### Isolation of the *Francisella* capsule

Bacteria were grown as a lawn for approximately 48 h on chocolate agar medium plates at 37°C and were collected by scraping colonies from the surface and suspending these in a solution of 6 mM Trizma base (Research Products International Corporation, Mt. Prospect, IL), 10 mM EDTA (Fisher Scientific, Fair Lawn, NJ) and 2.0% SDS (w/v) (Research Products International), pH 6.8 containing 50 µg/ml proteinase K and incubated at 65°C for 1 h and then overnight at 37°C. Additional Tris-SDS solution was added to completely solubilize the bacterial pellet after the 65°C incubation. The sample was placed at 37°C in a dry incubator overnight to complete the digestion. To remove the SDS, one tenth the volume of 0.3 M sodium acetate (0.1 ml per 1 ml sample) was added to the samples which were then precipitated with 3 volumes cold 100% ethanol, flash cooled in a dry ice-ethanol bath and incubated overnight at −20°C. Samples were centrifuged for 10 min at 12,000×*g* at 4°C and pellets were suspended in deionized water and precipitated a total of three times. Samples were suspended in 15 ml of 10 mM Tris pH 7.4 containing 10 mM CaCl_2_ and treated with 80 U micrococcal nuclease (Sigma, St. Louis, MO) overnight at 37°C. After incubation, an equal volume of 95% phenol was added to this mixture. Capsule samples and phenol were incubated at 65°C for 30 min, cooled on ice, and centrifuged at 2,000×*g* for 10 min at 4°C. The aqueous layer was collected and the phenol layer was back extracted with an equal volume of deionized water pre-warmed to 65°C. The aqueous layers were combined. To remove residual phenol, one-tenth volume of 0.3 M sodium acetate was added to the aqueous layers which were then precipitated three times with 3 volumes of absolute ethanol. The pellet was raised in distilled water and Triton X-114 (Sigma) was added to a final concentration of 5% (v/v). The sample was vortexed for 30 seconds and placed at 4°C. Sixteen hours later, the sample was placed at 37°C for two hours. The resulting detergent aqueous suspension was centrifuged at 2000×*g* at 37°C for ten minutes and the upper aqueous phase removed. The aqueous phase was dialyzed at room temperature against multiple changes of distilled water over 48 h and lyophilized. For some experiments, the lyophilized capsule was reconstituted in 6 mM Tris base, 10 mM EDTA and 2.0% SDS (w/v), pH 6.8 and placed over a 1×20 cm Sephacryl SH500 column equilibrated with the same buffer to remove residual LPS. The void volume of the column was 11 ml and the bed volume was 25 ml. The column was run at 37°C.

### Immunological assays

Western blots were performed as previously described using 4–12% gradient gels (Invitrogen, Carlsbad, CA) with samples transferred to PVDF (Millipore) [37]. ELISA assays were performed using CoStar 3590 plates (Corning Inc., Corning, NY) coated with 10 µg/ml of capsule as previously described [37].

### Cryoultramicrotomy for Immuno-TEM

*F. tularensis* colonies were removed intact using a punch and fixed in 4% paraformaldehyde with 0.2% glutaraldehyde in PBS overnight at 4°C. The plug was carefully trimmed into 1 mm square sections, treated with 2.3 M sucrose at 4°C overnight and then frozen by direct immersion in liquid nitrogen. Ultrathin frozen sections (70–95 nm) were cut on Leica EM UC6 Ultramicrotome and transferred onto a carbon-coated formvar covered nickel grid. These samples were then used for immunoelectron microscopy.

### Immunogold labeling for electron microscopy

The sections on the nickel grids were washed with PBS and then distilled water. They were blocked with 5% normal goat serum for 15 min. This was followed by incubation with the primary antibody, MAb 11B7 at a 1∶100 dilution in PBS for 30 min. The sample was washed with PBS thrice for 2 minutes each time. The secondary antibody was a goat anti-mouse 12 nm gold conjugate diluted 1∶40 in PBS and incubated on the sample at room temperature for 30 min. The grid was washed with PBS six times for 2 min each. As a final step, the grids were stained with 0.3% uranyl acetate in 2% methylcellulose for 8 min on ice. The excess solution was removed, the grid dried and the sample viewed with a JEOL 1230 TEM at an accelerating voltage of 120 kv.

Immunogold studies were also preformed on “whole mounted” bacteria with MAb 11B7 to evaluate surface labeling by the antibody directly. To accomplish this, bacteria were suspended in PBS and 5 µl were placed on a formvar coated nickel grid, the bacteria were allowed to settle for 10 minutes and excess fluid blotted off carefully with a Kimwipe. The sample was fixed by flooding the grid with a solution of 4% paraformaldehyde containing 0.05% ruthenium red. Excess fluid was removed with a Kimwipe. Five µl of MAb 11B7 at a dilution of 1∶500 was placed on the grid which was incubated at room temperature for 3 hours in a moist chamber. The grid was washed three times in PBS and a goat anti-mouse IgG 12 nm immunogold conjugate was placed on the grid for one hour. The grid was washed three times with PBS followed by distilled water. The grid was dried and viewed with a JEOL 1230 TEM at an accelerating voltage of 120 kv.

### Compositional analysis

For composition analyses capsule and LPS samples were run as alditol acetate (AA) and trimethylsilyl (TMS) derivatives. For AA derivatives, approximately 100 µg of sample was hydrolyzed to constituent monosaccharides with 4 N HCl at 100°C for 6 h. The acid was removed by flushing with dry nitrogen and re-evaporated using a 50% aqueous isopropanol solution. The monosaccharides were re-suspended in water and analyzed on a Dionex ICS-3000 equipped with a CarboPac PA-1 column, using a Pulsed Amperometric Detector (PAD). Samples were eluted using a sodium hydroxide and sodium acetate gradient. A standard set of monosaccharides were analyzed for comparison purposes. Alternatively, the monosaccharides were reduced to the corresponding alditols with either sodium borodeuteride or sodium borohydride overnight at room temperature. Reduced samples were neutralized with a 30% acetic acid solution and excess borates were removed by repeated co-evaporation with acidified methanol and anhydrous methanol several times. Finally, alditols were acetylated using a 1∶1 mixture of pyridine:acetic anhydride at 100°C for 1 h. Reagents were removed using a dry nitrogen flush. Samples were then extracted in dichloromethane and analyzed by GC-MS. For TMS derivatives, either 100 µg (capsule) or 300 µg (LPS) was methanolyzed using 1 M methanolic-HCl at 80°C for 16 h followed by removal of acidified methanol and re-*N*-acetylation using CH_3_OH:pyridine:acetic anhydride (5∶1∶1, by vol) at 100°C for 1 h. Reagents were removed by dry nitrogen flush, and the samples were silylated using Tri-Sil reagent at 80°C for 30 min. After removal of Tri-Sil by dry nitrogen flush, samples were extracted in hexane and analyzed by GC-MS. Composition analyses of capsule and LPS samples were performed on a Trace-MS Plus GC-MS (Thermo Fisher, Waltham, MA) equipped with an AS-2000 autosampler and a Rtx-5ms column (15 m×0.25 mm, df = 0.25 µm) operating in the electron impact (EI) mode. Two µl of sample was injected into the GC-MS operating in the split mode at 220°C, and the sample was split 1∶50. The initial oven temperature was set to 100°C, with a 5 min hold, followed by a ramping of the temperature at 4°C/min, with a final temperature of 270°C. Samples run in the chemical ionization mode (CI) were dissolved in dichloromethane and subsequently analyzed on a Varian GC-MS equipped with a DB-5 column (15 m×0.25 mm, df = 0.25 µm [Varian Inc., Palo Alto, CA]). One µl of sample was injected into the GC-MS, operating with a split ratio of 1∶25. The initial oven temperature was set to 120°C, with a 2 min hold, followed by a ramping of the temperature of 5°C/min, with a final temperature of 240°C, with a 5 min hold. Acetonitrile was used as the ionizing gas in CI mode.

### Linkage analysis

Linkage analysis of the capsule was done following a modified method of Ciucannu [38]. First, 0.4 mg of the sample was dissolved in 0.4 mL anhydrous dimethyl sulfoxide (DMSO) overnight, and then a slurry of sodium hydroxide in DMSO was added to the sample and incubated at room temperature for 1.5 h with constant stirring. Then, methyl iodide was added to the sample, followed by a second addition of methyl iodide 30 min later. The permethylated sample was then cooled, and extracted two times with chloroform:water (1∶2, v/v). The organic phase was dried and then hydrolyzed at 100°C for 6 h with 4 N trifluoroacetic acid. The sample was then reduced with sodium borodeuteride and acetylated with pyridine:acetic anhydride (1∶1) at 100°C for 6 h. Samples were then dried under a nitrogen stream, and the partially methylated alditol acetates (PMAA) were dissolved in dichloromethane and analyzed by GC-MS.

### MALDI-MS analyses of capsule

Unprocessed capsule was reconstituted in water at a concentration of 3 µg/µl. One µl of the sample was spotted onto a stainless steel MALDI-MS target, allowed to dry, and overlaid with 1 µl of a 50 mg/ml solution of 2,5 dihydroxybenzoic acid (DHB) (Laser Biolabs, Sophia-Antipolis Cedex, France) in 70% acetonitrile. Samples were subsequently analyzed using an LTQ linear ion trap mass spectrometer coupled to a vMALDI ion source (Thermo Fisher). The vMALDI source uses an SI N2 laser (337.3 nm) with a 20-Hz firing rate. Data was collected in the positive ion mode using the automated gain control (AGC) and the automatic spectrum filter (ASF). Tandem mass spectrometry (MS^n^) data were collected using a precursor ion selection window of 2 *m/z* and normalized collision energy of 35–40%. Alternatively, samples were analyzed in positive ion mode using the QStarXL equipped with an oMALDI source (Applied Biosystems, Foster City, CA).

### Mass spectrometric analyses of the capsule for associated lipids

To test for the presence of lipid A in the capsule, various preparations of capsule were generated and analyzed by mass spectrometry. Untreated LPS from *F. tularensis* strain 1547 was used for comparison purposes. The capsule was treated with hydrofluoric acid (HF) for either 2 hours at 20°C or 2 days at 4°C and subsequently dried down using nitrogen gas, under a vacuum, over sodium hydroxide. Samples were further processed by extraction with either CHCl_3_:CH_3_OH:H_2_O (10∶5∶6) or CHCl_3_:CH_3_OH (1∶1), respectively. The organic phases and precipitate were evaporated to dryness under a nitrogen stream. Untreated samples were reconstituted in water, spotted onto a MALDI target, dried, and overlaid with DHB matrix (50 mg/ml in 70% acetonitrile). Chloroform extracted samples were reconstituted in CHCl_3_:CH_3_OH (3∶1) and overlaid with 6-chloro-3-mercaptobenzothiazole (CMBT) matrix (saturated solution in CHCl_3_:CH_3_OH (3∶1). Samples were analyzed using an LTQ linear ion trap mass spectrometer coupled to a vMALDI ion source (Thermo Fisher) operating in the negative ion mode using the conditions listed above.

### NMR methods

An NMR sample was prepared by lyophilizing 4 mg of purified *F. tularensis* capsule polysaccharide against 3 changes of D_2_O and dissolving the dried material in 0.5 mL of 99.96% D_2_O from a freshly broken ampoule. All spectra were acquired at 35°C on a 600 MHz Varian UnityInova NMR spectrometer equipped with a standard triple resonance probe with pulsed field gradients except for an HMBC experiment which was acquired on an 800 MHz Bruker Avance II NMR spectrometer equipped with a TCI cryoprobe. All spectra were processed using NMRPipe [39], [40] and analyzed using Sparky. The following datasets were used for analysis: ^13^C-HMQC, gHMBC, gCOSY, DQF-COSY, TOCSY (20 ms and 60 ms mixing times), and NOESY (200 ms mixing time). Acquisition and processing parameters are shown in Table 6.

**Table 6:** NMR Spectral Parameters.

Experiment	F1 SW (Hz)	F2 SW (Hz)	F1 points, acquired	F2 points, acquired	F1 points, processed	F2 points, processed
1H-13C HMQC	7199.4	12062.7	512*	140*	1024	512
DQF-COSY	5205.6	5205.6	1024*	620*	4096	4096
gCOSY	5205.6	2505.6	2048*	1024	4096	4096
gHMBC	11029.4	38314.2	2048*	700	4096	2048
TOCSY, 20 ms	7199.4	7199.4	1024*	256*	4096	2048
TOCSY, 60 ms	7199.4	7199.4	1024*	256*	4096	2048
NOESY, 200 ms	7199.4	7199.4	512*	256*	2048	2048

#### Ethics Statements

All animals were handled in strict accordance with good animal practice as defined by the relevant national and/or local animal welfare bodies, and all animal work was approved by the University of Iowa Animal Care and Use committee (ACURF # 0808184). Guidelines provided by the NIH were followed in all experimentation. The University of Iowa is PHS assured.

### Passive immunization with MAb 11B7 of BALB/c mice and challenge with *F. tularensis* LVS

BALB/c female mice 6–8 weeks of age were purchased from NCI. Two groups of five mice were injected intraperitoneally with either 75 µg of 11B7 antibody or 75 µg of a matched isotype monoclonal antibody, 2C3, as a negative control. Antibody 2C3 binds to a *Neisseria gonorrhoeae* membrane protein, H.8. Both MAbs, 11B7 and 2C3, are IgG1κmonoclonal antibodies. MAb 2C3 was a gift from Dr. Peter Rice, University of Massachusetts. Both antibodies were affinity purified over a Protein G (Thermo Scientific, Rockford, IL) column according to manufacturer's instructions. The protein concentration was determined using an Easy Titer Mouse IgG assay Kit (Thermo Scientific, Rockford, IL). Twenty four hours post-injection of antibody, both groups of 5 mice were challenged intraperitoneally with 5×10^4^ cfu (∼100 LD_50_) of *F. tularensis* LVS and the ability of the mice to survive challenge was followed for 14 days. A second experiment was performed using the same protocol for administration of monoclonal antibodies but the challenge dose of *F. tularensis* LVS was 5×10^5^ cfu (∼1000 LD_50_).

### Challenge of BALB/c mice with *F. tularensis* LVS previously immunized with capsule

BALB/c female mice 6–8 weeks of age were purchased from NCI. Groups of 5 mice were used for each immunization and challenge protocol. Two groups of five mice were injected intraperitoneally with 50 µl of phosphate buffer saline (PBS) and TiterMax Gold adjuvant at a 1∶1 ratio. Two separate groups of mice were immunized intraperitoneally with 10 µg of capsule in a 25 µl volume mixed 1∶1 with 25 µl TiterMax Gold adjuvant. Mice were bled retro-orbitally 30 days post-immunization to test for the presence of anti-capsule antibodies in the serum. One day post bleeding each group of mice was given a booster dose of antigen and adjuvant identical to the original immunization dose. Two weeks after the boost dose, the mice were bled again and the anti- capsule antibody was re-assessed. Forty eight hours post-bleeding, the groups of mice immunized with PBS, were challenged intraperitoneally with 250 and 750 cfu of *F. tularensis* LVS. One group of mice, immunized with 10 µg of capsule in adjuvant, was challenged intraperitoneally with 5×10^4^ LVS and the other group of mice immunized with 10 µg of capsule in adjuvant was challenged intraperitoneally with 5×10^5^ cfu of LVS. All mice were followed for 14 days to determine the ability of the mice to survive the challenge.

#### Statistical Analysis

For the challenge experiments, Kaplan-Meier survival estimates [41] of the survival of each group of mice were calculated and differences between groups tested using a log-rank test statistic [41], [42]. Survival of all control mice were compared to survival of all mice treated with MAb 11B7, and each control group was compared to all immunized mice. All calculations were done in R (R Development Core Team, 2009) and the R survival package [43], [44].

## Supporting Information

Figure S1 Figure S1 shows a Western Blot that demonstrates that capsule does not appear to be shed during growth into the supernate of liquid cultures. One microgram of each of the following underwent SDS-PAGE using a 4–12% gel followed by transfer to nitrocellulose. Lane 1 contains the molecule weight controls and the arrow indicates 200 kDa, lane 2 is an organism pellet loaded into sample buffer, lane 3 is purified LVS capsule, lane 4, 5 and 6 are 70% ethanol precipitates of 4 (lane 5), 8 (lane 6) and 24 (lane 7) hour supernates cleared of organisms by centrifugation at 13,500×g. The bacterial pellets removed from the 4, 8 and 24 hour supernates are in lanes 8, 9 and 10 respectively. Lane 4 and 11 contained no sample. Lane 12 contains an TX-114 extracted LPS sample. The Western blot was developed with MAb 11B7 at a dilution of 1;10,000. These studies show that the majority of the LVS capsule is associated with the organism and only minimal amounts of capsule are released into the broth supernate at 24 hours.
(1.88 MB TIF)

Figure S2 Figure S2 shows the positive-ion MALDI-TOF mass spectra of unprocessed capsule. The predominant monoisotopic mass observed is m/z 815, this mass corresponds to the sodiated form of the 792 Da tetrasaccharide repeating unit. These data show that we were able to detect up to six repeating units in the capsule sample.
(0.21 MB TIF)

Figure S3 Figure S3 shows the positive-ion vMALDI-LIT mass spectra of HF-treated capsule. After HF treatment, the predominant monoisotopic sodiated masses observed are at m/z 833 and 1625 which corresponds to one or two units of the 792 Da tetrasaccharide repeat, respectively. The fragments observed after HF treatment were generated by chemical hydrolysis and therefore contain an additional water (793+18 = 811 Da) relative to the peaks observed in the unprocessed capsule sample that were generated by gas-phase fragmentation. Masses labeled with an * designate major masses minus water.
(0.23 MB TIF)

Figure S4 Figure S4 shows the positive ion-vMALDI-MS^n^ analysis of unprocessed capsule. The tetrasaccharide at m/z 793 (A) was sequentially fragmented to yield fragment ions at m/z 606 (B), at m/z 433(C), and at m/z 390 (D). The MS^4^ data of m/z 433 demonstrated that it is composed of two carbohydrates monomers of the same mass, 216 Da, that are adjacent to one another. The MS^4^ data of m/z 390 demonstrated that it is composed of two carbohydrate monomers with masses of 173 Da and 216 Da that are located adjacent to one another. Masses labeled with an * designate major masses +/− water.
(0.65 MB TIF)

Figure S5 Figure S5 shows the positive ion-vMALDI-MS^n^ analyses of unprocessed capsule. The tetrasaccharide at m/z 793 (A) was sequentially fragmented to yield fragment ions at m/z 577 (B) and at m/z 361 (C). The MS^4^ data of m/z 361 demonstrated that it is composed of two carbohydrate monomers with masses of 173 Da and 187 Da that are located adjacent to one another. Masses labeled with an * designate major masses +/− water.
(0.46 MB TIF)

Figure S6 Figure S6 shows the (A) total ion chromatogram of alditol acetate (AA) derivatives of F. tularensis capsule. Three major peaks were observed. One of these peaks could be assigned to QuiNAc (RT 23.56 min), based on CI (B) and EI (C) data. The two mass fragments observed in EI mode at m/z 302 and 144 CI are consistent with the QuiNAc assignment. EI analyses suggested that the unknown peaks are most likely anhydro-degradation products of QuiNAc or deformylated Qui4NFm. No peak was observed that was consistent with HexNAcAN; it has been previously suggested that this sugar is either too labile or polar to be observed by these analyses (Y. A. Knirel, et al. Eur. J. Biochem. 1985, 150:541-550). Inositol was spiked in as a control.
(0.53 MB TIF)

Figure S7 Figure S7 shows (A) the total ion chromatogram of alditol acetate (AA) derivatives of F. tularensis LPS. QuiNAc, GlcNAc, GalNAc. Two unknown peaks were detected in these samples. CI (B) and EI (C) analyses verified the presence of QuiNAc. The two mass fragments observed in EI mode at m/z 302 and 144 CI are consistent with the QuiNAc assignment. CI and EI analsyses also suggested that the unknown peaks are most likely ahydro degradation products of QuiNAc or deformylated Qui4NFm. Inositol was spiked in as a control.
(0.64 MB TIF)

Figure S8 Figure S8 shows the total ion chromatogram of trimethylsilyl (TMS) derivative of the capsule sample (A). QuiNAc and HexNAcAN were detected in these samples. Fragmentation analysis verified their identification (B and C, respectively). Mannitol was included as a control.
(1.22 MB TIF)

Figure S9 Figure S9 shows the (A) Total ion chromatogram of TMS derivative of the F. tularensis LPS scanned over a mass range of m/z 50–600. These data show that the main constituents observed in this sample include lipid A components as well as core sugars. (B) Selective ion chromatogram of the LPS sample to scan for amino sugars. The sample was scanned over the mass range of m/z 172.5–173.5. These data confirmed the presence of QuiNAc, GalNAc, and GlcNAc.
(0.50 MB TIF)

Figure S10 Figure S10 shows the total ion chromatogram of partially methylated alditol acetate (PMAA) derivatives of F. tularensis capsule. Data was collected over a mass range of m/z 50–600. QuiNAc with a 3-linkage and a terminal linkage were identified at 25.40 and 22.57 min, respectively. Fragmentation analysis verified these assignments (B and C, respectively).
(0.61 MB TIF)

Table S1 Proposed compositions of masses observed by MALDI-TOF analyses.
(0.03 MB DOC)

Table S2 High pH-chromatography data from mixture of standards.
(0.04 MB DOC)

Table S3 High pH-chromatography data from F. tularensis capsule.
(0.04 MB DOC)

Table S4 Summary of GC-MS data for TMS derivatives of F. tularensis capsule.
(0.03 MB DOC)

Table S5 Summary of GC-MS data for TMS derivatives of F. tularensis LPS.
(0.03 MB DOC)
