# Supplementary figures and images for: Identification, Characterization and Immunogenicity of an O-Antigen Capsular Polysaccharide of Francisella tularensis
Source: PLoS One. 2010 Jul 6;5(7):e11060. doi: 10.1371/journal.pone.0011060 (PMC2897883; doi:10.1371/journal.pone.0011060)

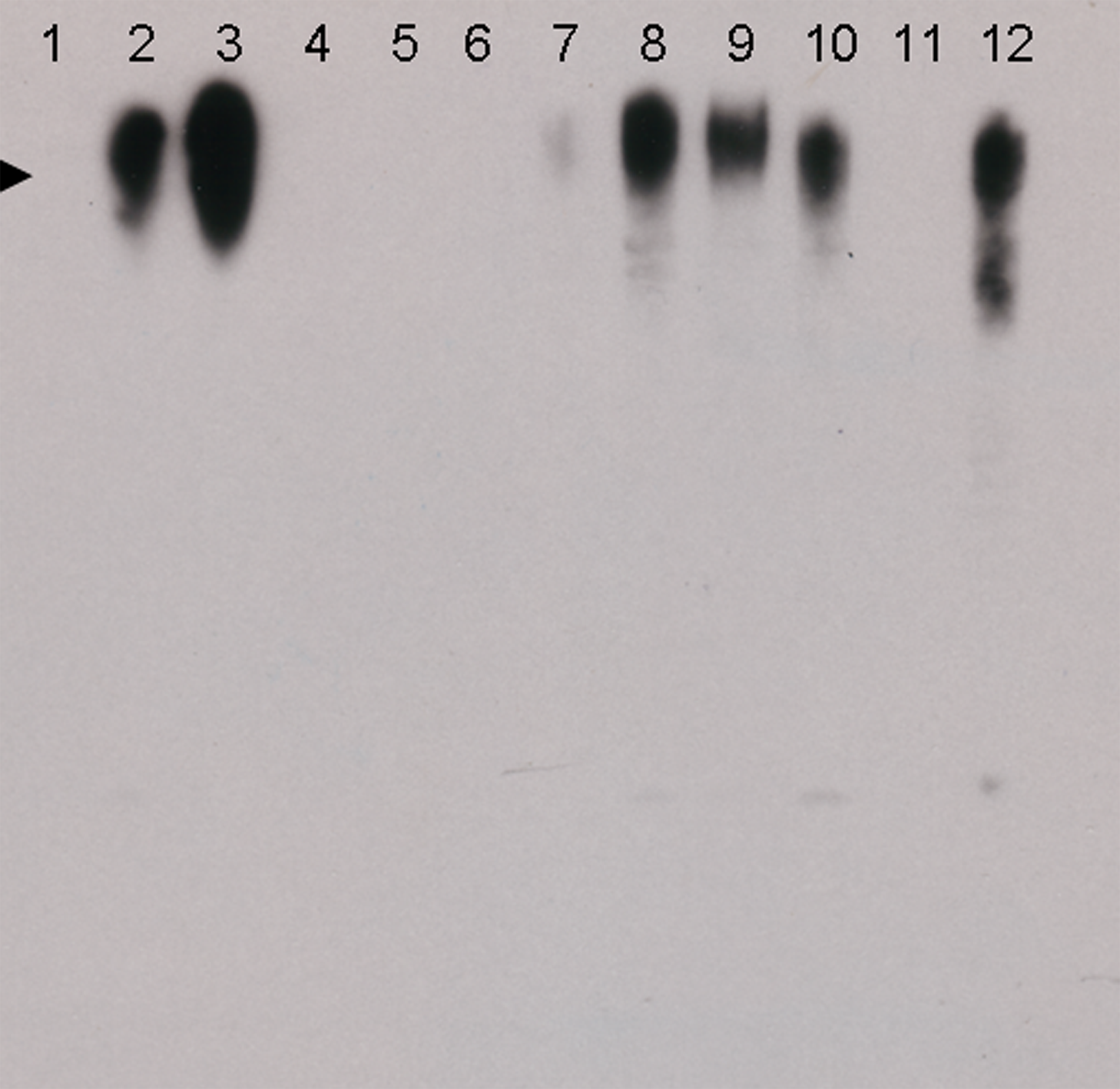

Supplement: Figure S1 — shows a Western Blot that demonstrates that capsule does not appear to be shed during growth into the supernate of liquid cultures. One microgram of each of the following underwent SDS-PAGE using a 4–12% gel followed by transfer to nitrocellulose. Lane 1 contains the molecule weight controls and the arrow indicates 200 kDa, lane 2 is an organism pellet loaded into sample buffer, lane 3 is purified LVS capsule, lane 4, 5 and 6 are 70% ethanol precipitates of 4 (lane 5), 8 (lane 6) and 24 (lane 7) hour supernates cleared of organisms by centrifugation at 13,500×g. The bacterial pellets removed from the 4, 8 and 24 hour supernates are in lanes 8, 9 and 10 respectively. Lane 4 and 11 contained no sample. Lane 12 contains an TX-114 extracted LPS sample. The Western blot was developed with MAb 11B7 at a dilution of 1;10,000. These studies show that the majority of the LVS capsule is associated with the organism and only minimal amounts of capsule are released into the broth supernate at 24 hours. (1.88 MB TIF) [file pone.0011060.s001.tif]

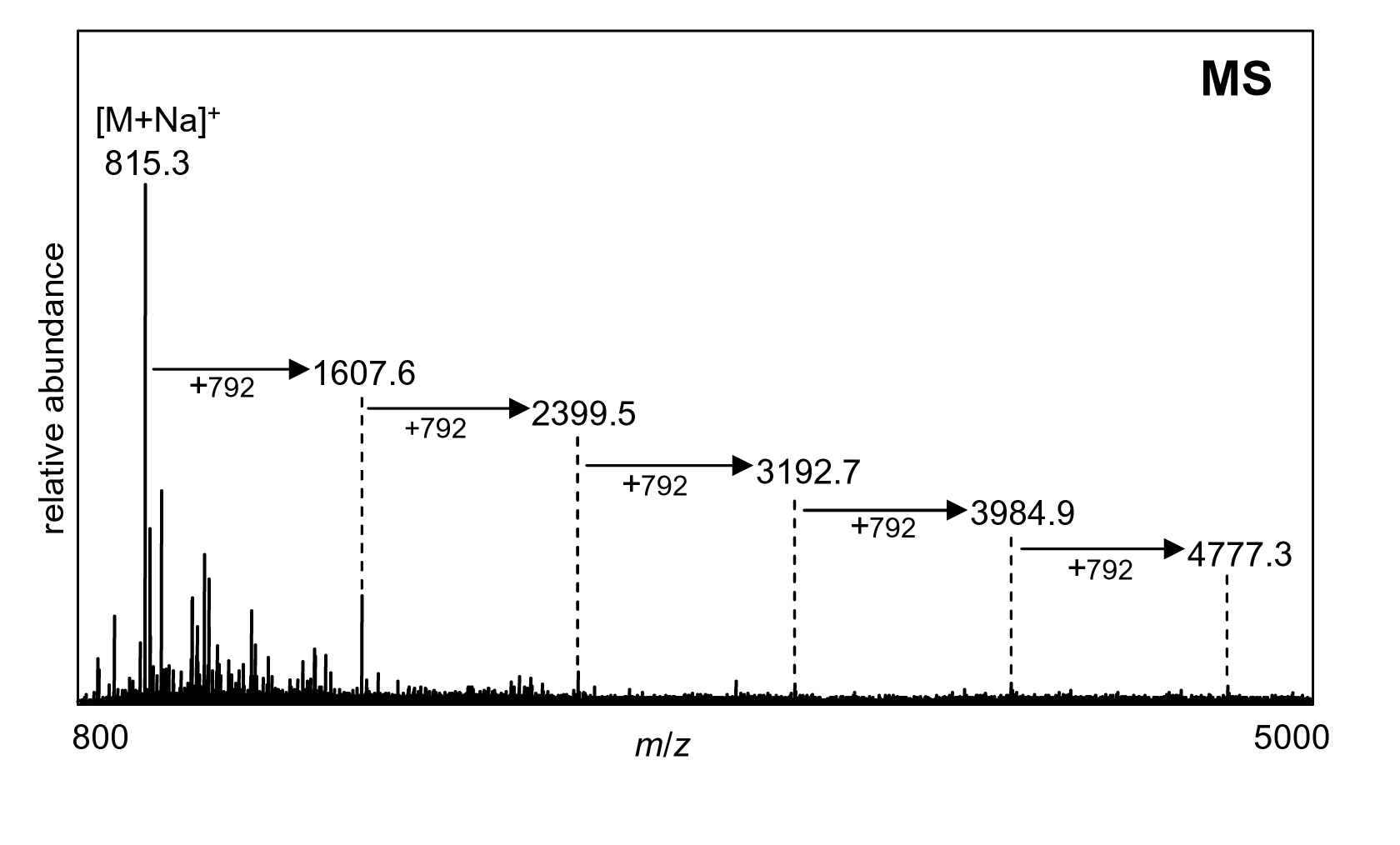

Supplement: Figure S2 — shows the positive-ion MALDI-TOF mass spectra of unprocessed capsule. The predominant monoisotopic mass observed is m/z 815, this mass corresponds to the sodiated form of the 792 Da tetrasaccharide repeating unit. These data show that we were able to detect up to six repeating units in the capsule sample. (0.21 MB TIF) [file pone.0011060.s002.tif]

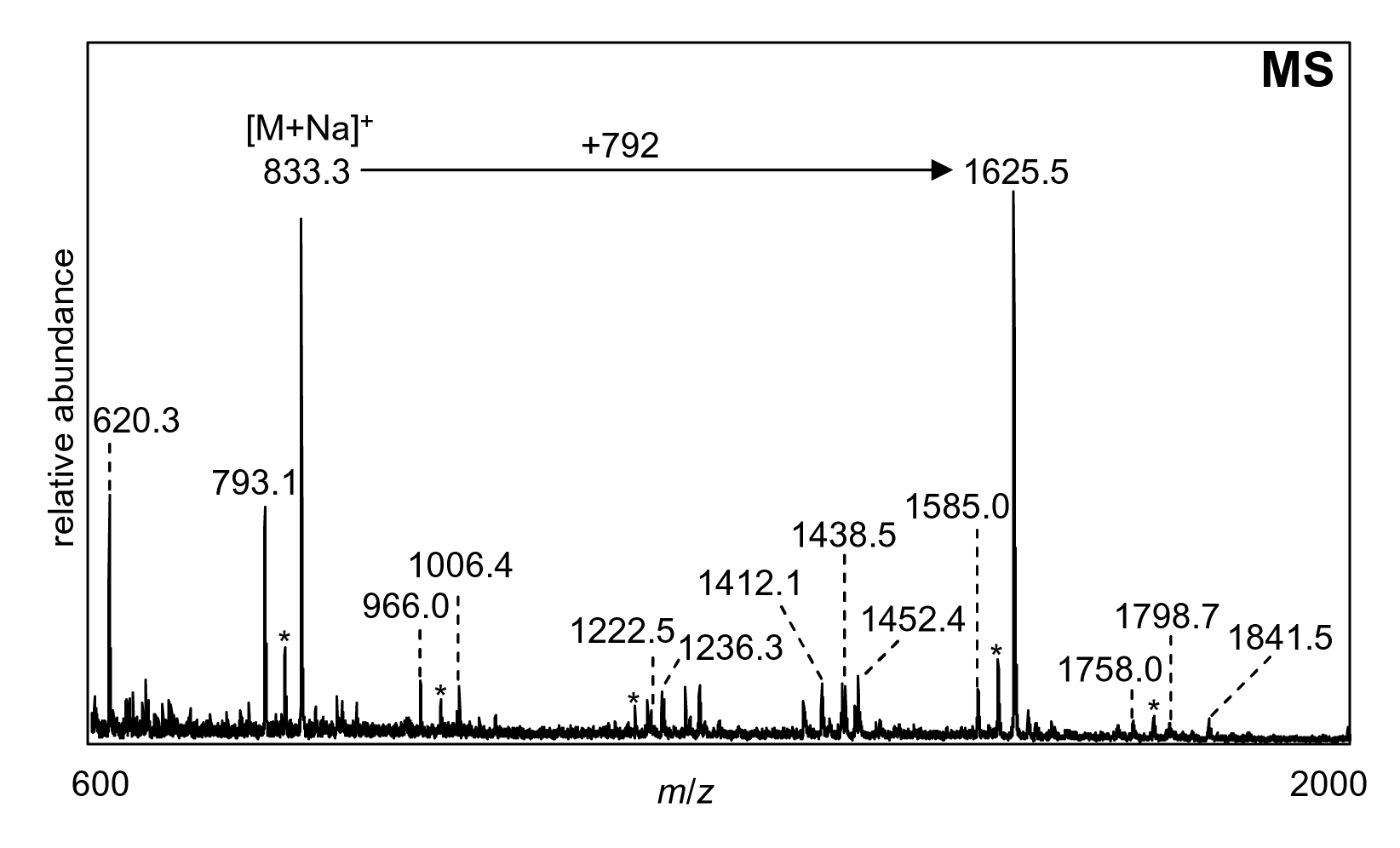

Supplement: Figure S3 — shows the positive-ion vMALDI-LIT mass spectra of HF-treated capsule. After HF treatment, the predominant monoisotopic sodiated masses observed are at m/z 833 and 1625 which corresponds to one or two units of the 792 Da tetrasaccharide repeat, respectively. The fragments observed after HF treatment were generated by chemical hydrolysis and therefore contain an additional water (793+18 = 811 Da) relative to the peaks observed in the unprocessed capsule sample that were generated by gas-phase fragmentation. Masses labeled with an * designate major masses minus water. (0.23 MB TIF) [file pone.0011060.s003.tif]

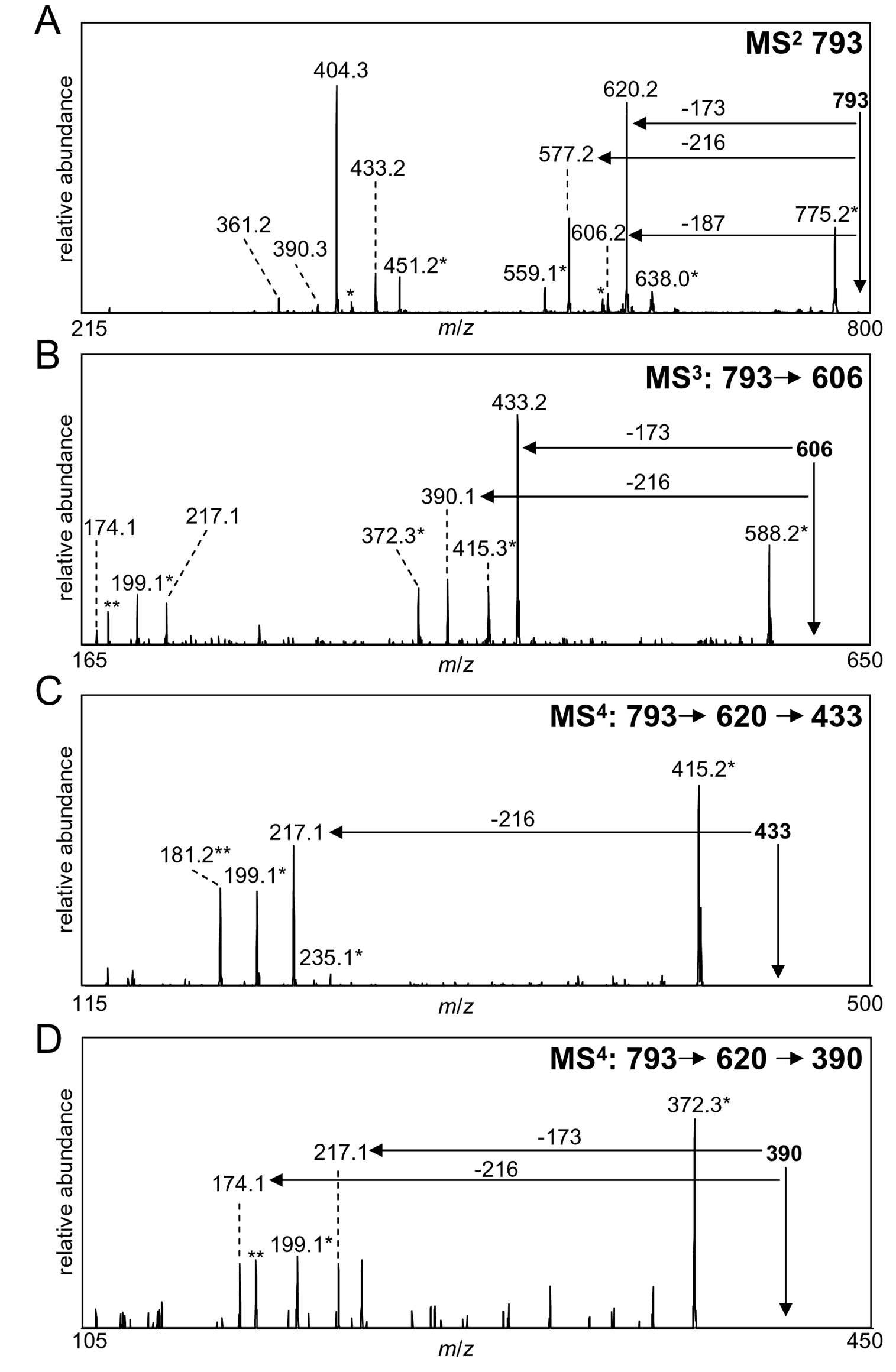

Supplement: Figure S4 — shows the positive ion-vMALDI-MSn analysis of unprocessed capsule. The tetrasaccharide at m/z 793 (A) was sequentially fragmented to yield fragment ions at m/z 606 (B), at m/z 433(C), and at m/z 390 (D). The MS4 data of m/z 433 demonstrated that it is composed of two carbohydrates monomers of the same mass, 216 Da, that are adjacent to one another. The MS4 data of m/z 390 demonstrated that it is composed of two carbohydrate monomers with masses of 173 Da and 216 Da that are located adjacent to one another. Masses labeled with an * designate major masses +/− water. (0.65 MB TIF) [file pone.0011060.s004.tif]

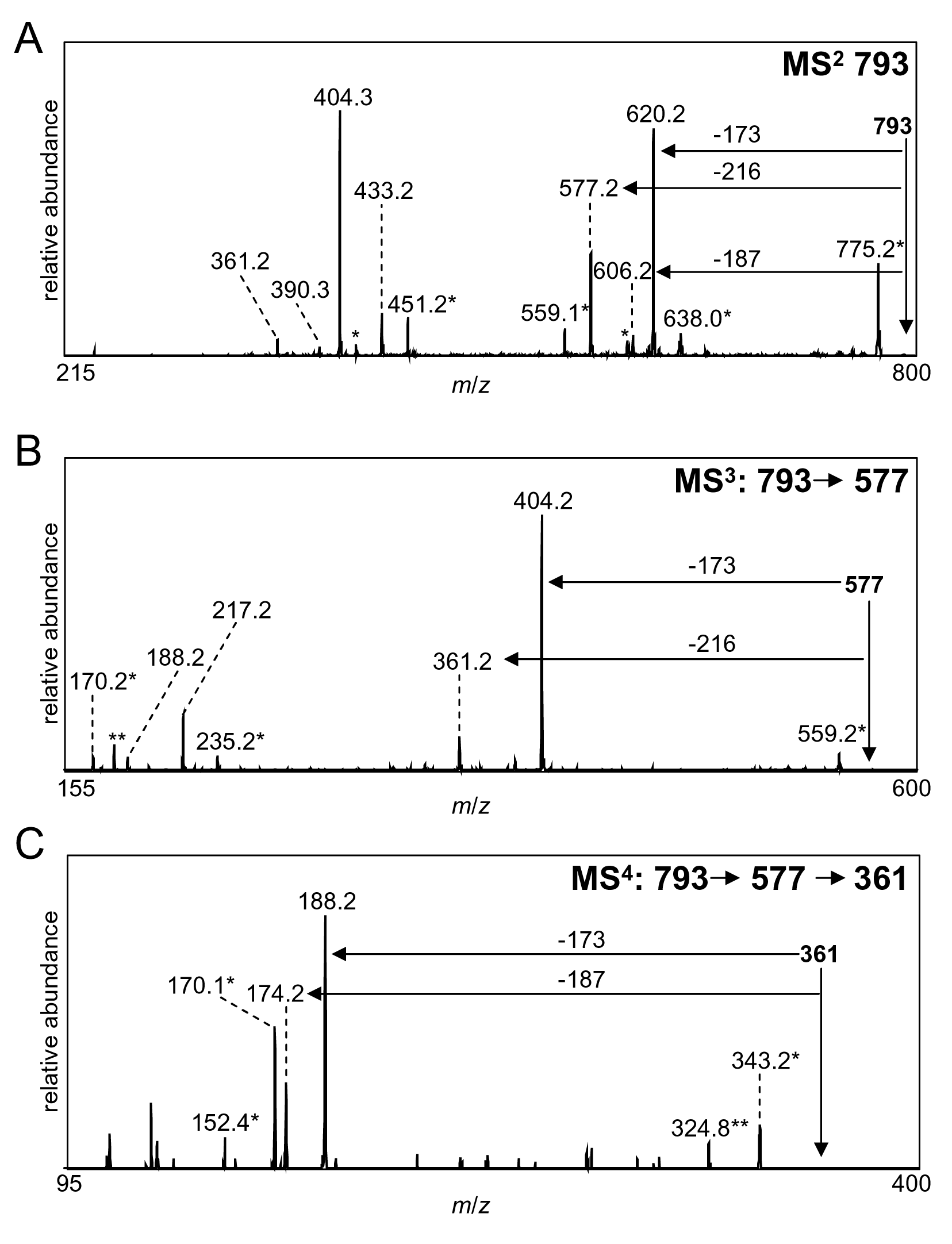

Supplement: Figure S5 — shows the positive ion-vMALDI-MSn analyses of unprocessed capsule. The tetrasaccharide at m/z 793 (A) was sequentially fragmented to yield fragment ions at m/z 577 (B) and at m/z 361 (C). The MS4 data of m/z 361 demonstrated that it is composed of two carbohydrate monomers with masses of 173 Da and 187 Da that are located adjacent to one another. Masses labeled with an * designate major masses +/− water. (0.46 MB TIF) [file pone.0011060.s005.tif]

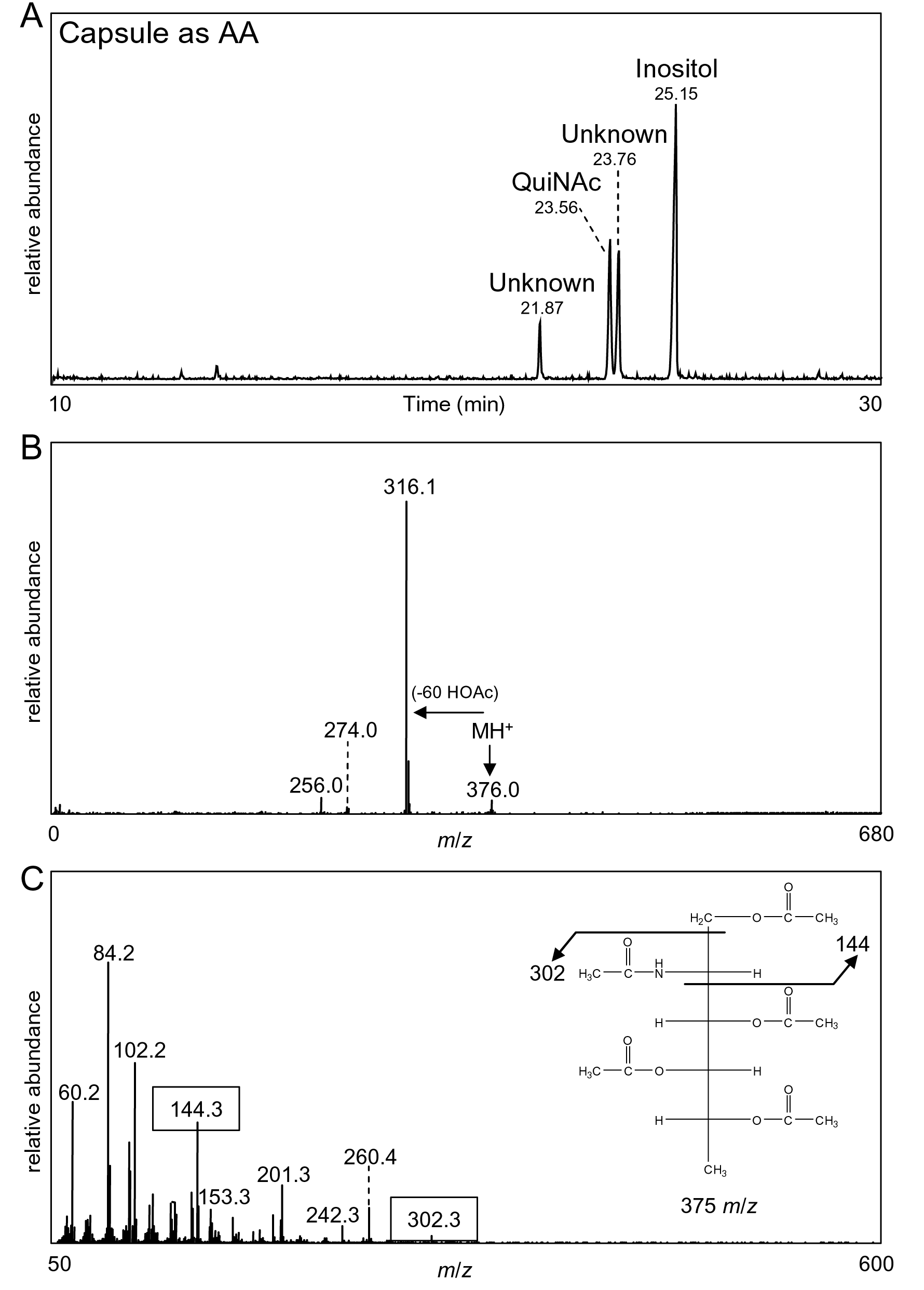

Supplement: Figure S6 — shows the (A) total ion chromatogram of alditol acetate (AA) derivatives of F. tularensis capsule. Three major peaks were observed. One of these peaks could be assigned to QuiNAc (RT 23.56 min), based on CI (B) and EI (C) data. The two mass fragments observed in EI mode at m/z 302 and 144 CI are consistent with the QuiNAc assignment. EI analyses suggested that the unknown peaks are most likely anhydro-degradation products of QuiNAc or deformylated Qui4NFm. No peak was observed that was consistent with HexNAcAN; it has been previously suggested that this sugar is either too labile or polar to be observed by these analyses (Y. A. Knirel, et al. Eur. J. Biochem. 1985, 150:541-550). Inositol was spiked in as a control. (0.53 MB TIF) [file pone.0011060.s006.tif]

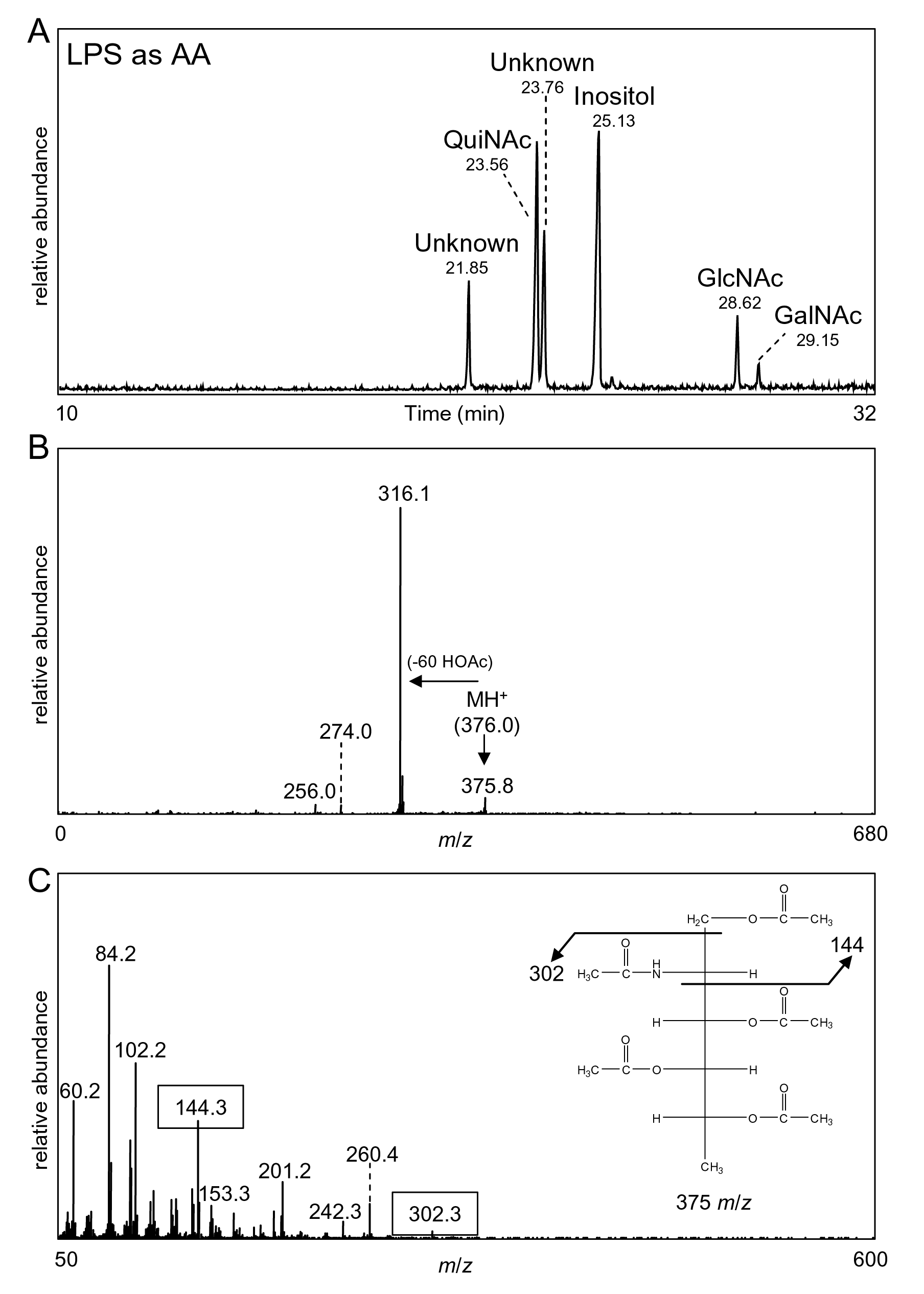

Supplement: Figure S7 — shows (A) the total ion chromatogram of alditol acetate (AA) derivatives of F. tularensis LPS. QuiNAc, GlcNAc, GalNAc. Two unknown peaks were detected in these samples. CI (B) and EI (C) analyses verified the presence of QuiNAc. The two mass fragments observed in EI mode at m/z 302 and 144 CI are consistent with the QuiNAc assignment. CI and EI analsyses also suggested that the unknown peaks are most likely ahydro degradation products of QuiNAc or deformylated Qui4NFm. Inositol was spiked in as a control. (0.64 MB TIF) [file pone.0011060.s007.tif]

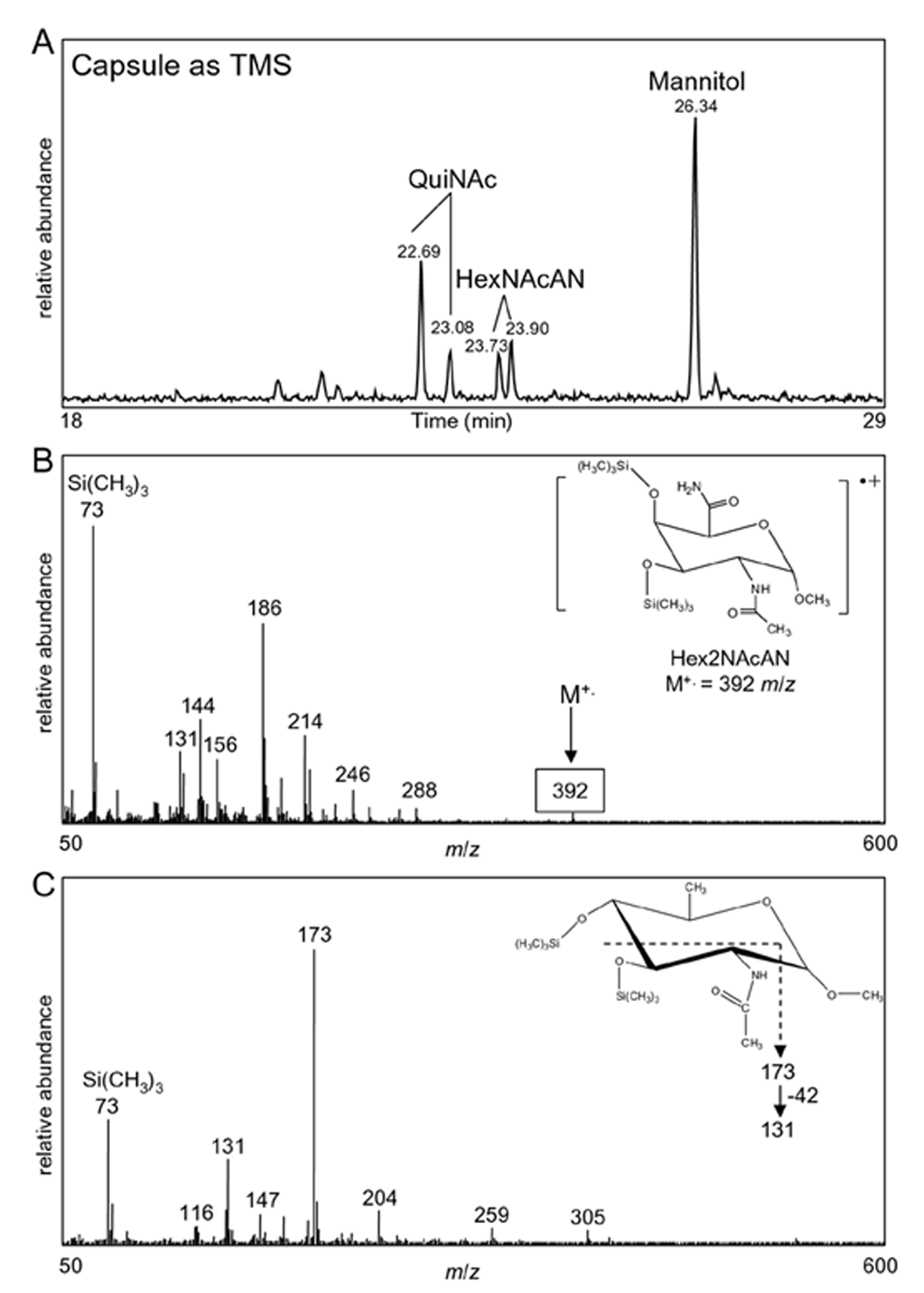

Supplement: Figure S8 — shows the total ion chromatogram of trimethylsilyl (TMS) derivative of the capsule sample (A). QuiNAc and HexNAcAN were detected in these samples. Fragmentation analysis verified their identification (B and C, respectively). Mannitol was included as a control. (1.22 MB TIF) [file pone.0011060.s008.tif]

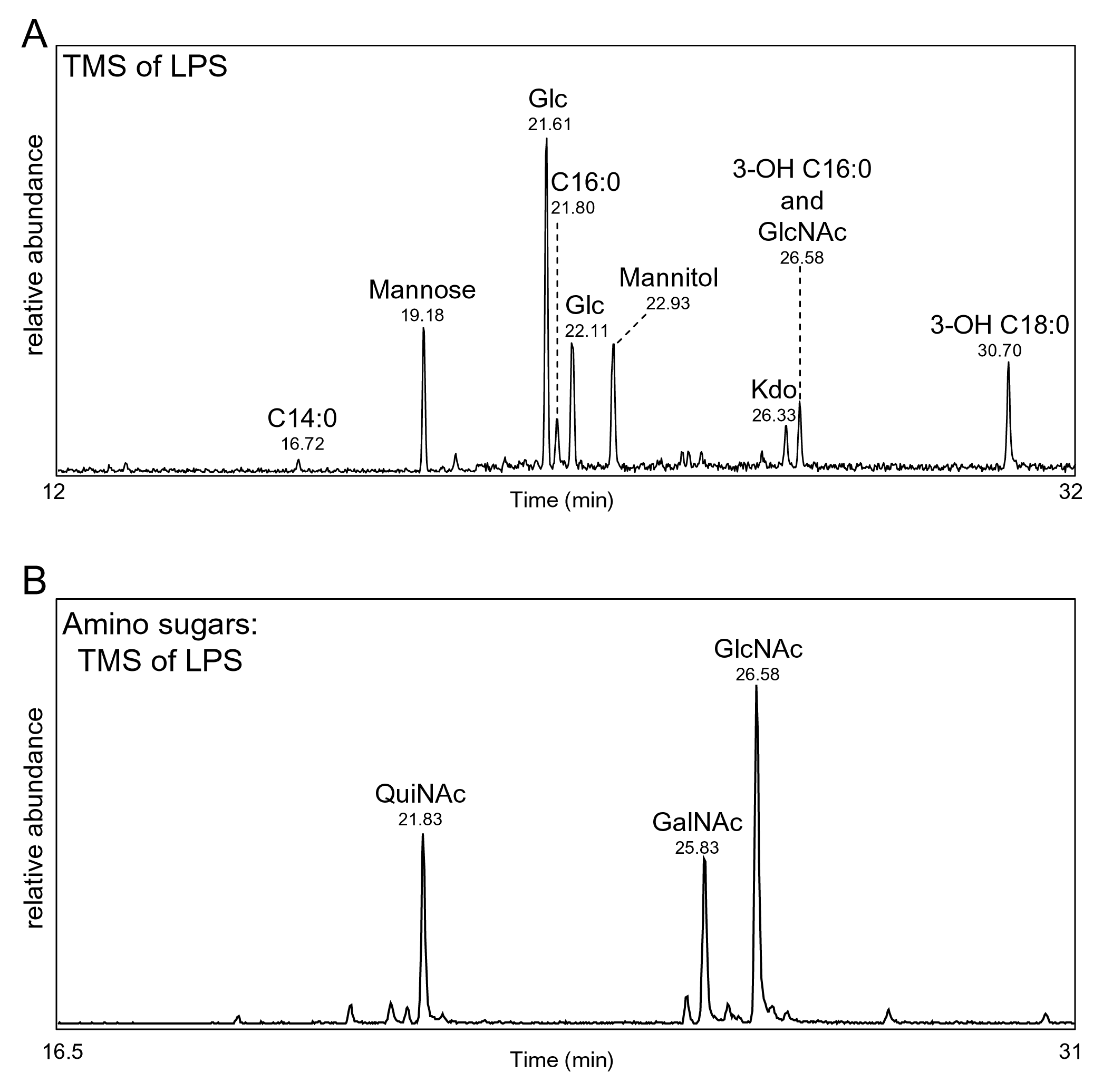

Supplement: Figure S9 — shows the (A) Total ion chromatogram of TMS derivative of the F. tularensis LPS scanned over a mass range of m/z 50–600. These data show that the main constituents observed in this sample include lipid A components as well as core sugars. (B) Selective ion chromatogram of the LPS sample to scan for amino sugars. The sample was scanned over the mass range of m/z 172.5–173.5. These data confirmed the presence of QuiNAc, GalNAc, and GlcNAc. (0.50 MB TIF) [file pone.0011060.s009.tif]

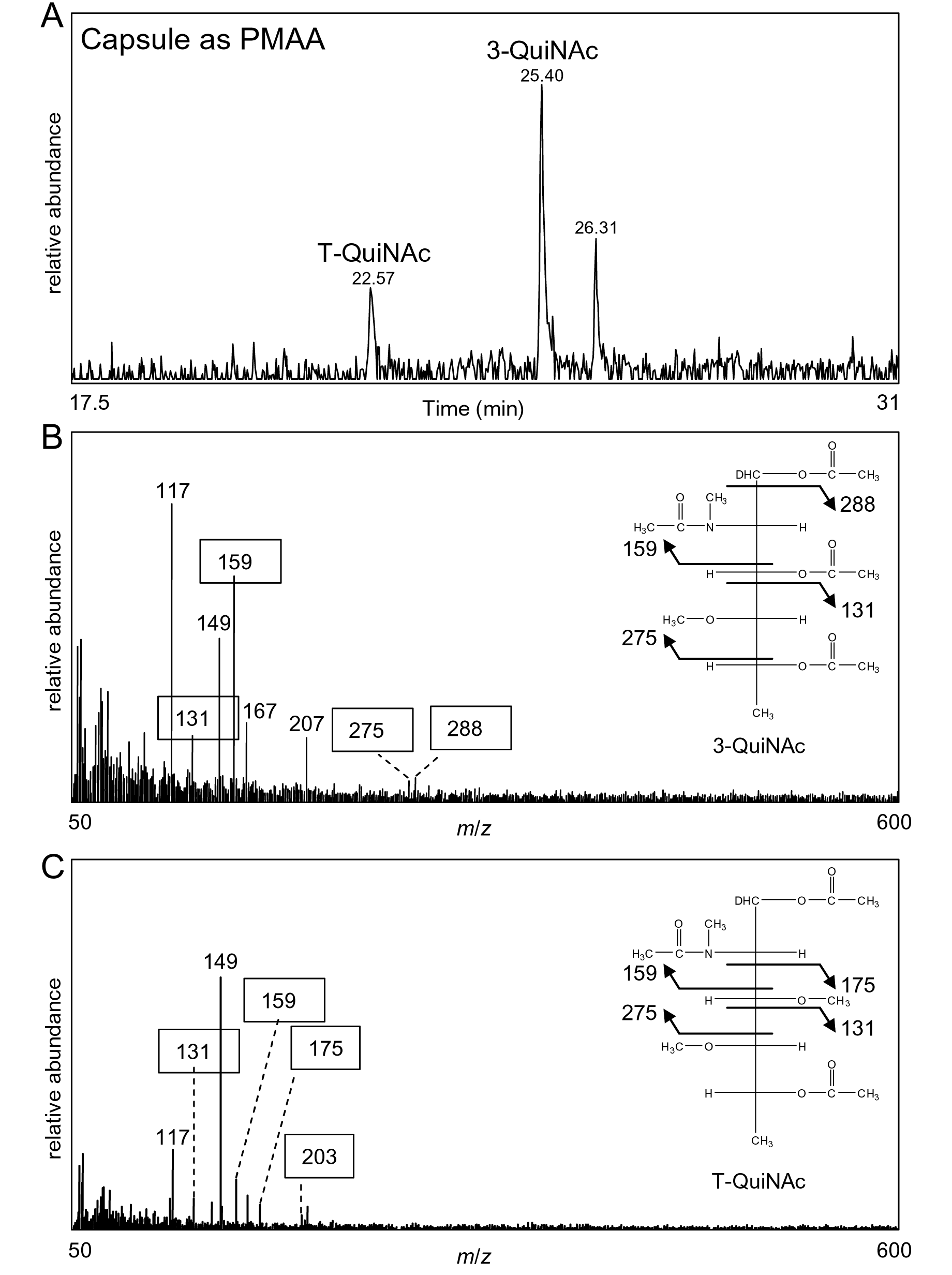

Supplement: Figure S10 — shows the total ion chromatogram of partially methylated alditol acetate (PMAA) derivatives of F. tularensis capsule. Data was collected over a mass range of m/z 50–600. QuiNAc with a 3-linkage and a terminal linkage were identified at 25.40 and 22.57 min, respectively. Fragmentation analysis verified these assignments (B and C, respectively). (0.61 MB TIF) [file pone.0011060.s010.tif]
