# Supplementary material for: Identification, Characterization and Immunogenicity of an O-Antigen Capsular Polysaccharide of Francisella tularensis
Source: PLoS One. 2010 Jul 6;5(7):e11060. doi: 10.1371/journal.pone.0011060 (PMC2897883; doi:10.1371/journal.pone.0011060)
Supplement: Table S1 — Proposed compositions of masses observed by MALDI-TOF analyses. (0.03 MB DOC) [file pone.0011060.s011.doc]

| [M+Na]^+^_observed_ | [M+Na]^+^_calculated_ | Proposed composition^1^ |
| --- | --- | --- |
| 815.32 | 815.31 | (GalNAcAN-GalNAcAN-QuiNAc-Qui4NFm)_1_ |
| 1607.56 | 1607.63 | (GalNAcAN-GalNAcAN-QuiNAc- Qui4NFm)_2_ |
| 2399.47 | 2399.95 | (GalNAcAN-GalNAcAN-QuiNAc- Qui4NFm)_3_ |
| 3192.66 | 3192.27 | (GalNAcAN-GalNAcAN-QuiNAc- Qui4NFm)_4_ |
| 3984.88 | 3984.59 | (GalNAcAN-GalNAcAN-QuiNAc- Qui4NFm)_5_ |
| 4777.29 | 4776.91 | (GalNAcAN-GalNAcAN-QuiNAc- Qui4NFm)_6_ |

**Table S1**: Proposed compositions of masses observed by MALDI-TOF analyses

^1^GalNAcAN: 2-acetamido-2-deoxy-D-galacturonamide (M= 216.08 Da), QuiNAc: 2-acetamido-2,6-dideoxy-D-glucose (M= 187.09 Da), and Qui4NFm: 4,6-dideoxy-4-formamido-D-glucose (M= 173.07 Da). Masses listed are anhydro forms of each constituent.
