# Supplementary material for: Identification, Characterization and Immunogenicity of an O-Antigen Capsular Polysaccharide of Francisella tularensis
Source: PLoS One. 2010 Jul 6;5(7):e11060. doi: 10.1371/journal.pone.0011060 (PMC2897883; doi:10.1371/journal.pone.0011060)
Supplement: Table S2 — High pH-chromatography data from mixture of standards. (0.04 MB DOC) [file pone.0011060.s012.doc]

**Table S2**: High pH-chromatography data from mixture of standards.

| Peak name | Ret. time (min) | Height  (nC) | Area  (nC*min) | Relative Area  (%) |
| --- | --- | --- | --- | --- |
| Fucose | 5.08 | 41.395 | 11.522 | 6.41 |
| Galactosamine | 10.17 | 74.981 | 36.301 | 20.19 |
| Glucosamine | 12.17 | 45.043 | 21.129 | 11.75 |
| Galactose | 13.42 | 25.916 | 11.888 | 6.61 |
| Glucose | 14.50 | 40.288 | 20.991 | 11.67 |
| Mannose | 16.08 | 13.781 | 9.973 | 5.55 |
| *N*-acetylneuraminic acid | 43.67 | 44.717 | 14.494 | 8.06 |
| ^1^Kdo | 47.67 | 24.207 | 8.790 | 4.89 |
| Galacturonic acid | 52.50 | 5.459 | 1.847 | 1.03 |
| Glucuronic acid | 54.50 | 19.809 | 7.402 | 4.12 |
| *N*-glycolylneuraminic acid | 59.08 | 65.544 | 29.596 | 16.46 |
| Iduronic acid | 61.17 | 11.753 | 5.893 | 3.28 |

^1^Kdo: 3-deoxy-D-*manno*-octulosonic acid
