# Supplementary material for: Identification, Characterization and Immunogenicity of an O-Antigen Capsular Polysaccharide of Francisella tularensis
Source: PLoS One. 2010 Jul 6;5(7):e11060. doi: 10.1371/journal.pone.0011060 (PMC2897883; doi:10.1371/journal.pone.0011060)
Supplement: Table S3 — High pH-chromatography data from F. tularensis capsule. (0.04 MB DOC) [file pone.0011060.s013.doc]

| Peak name | Ret. time (min) | Height  (nC) | Area  (nC*min) | Relative Area  (%) |
| --- | --- | --- | --- | --- |
| Unknown | 4.17 | 120.134 | 108.367 | 18.43 |
| Unknown | 6.83 | 444.836 | 179.051 | 30.46 |
| Galactosamine | 10.17 | 22.212 | 8.765 | 1.49 |
| Glucosamine | 12.25 | 28.760 | 13.650 | 2.32 |
| Glucose | 14.50 | 15.189 | 7.612 | 1.29 |
| Unknown | 46.50 | 58.080 | 19.053 | 3.24 |
| Unknown | 50.00 | 534.195 | 219.448 | 37.33 |
| Unknown | 63.33 | 16.599 | 6.657 | 1.13 |
| Unknown | 69.58 | 51.572 | 25.270 | 4.30 |

| Peak name | Ret. time (min) | Height  (nC) | Area  (nC*min) | Relative Area  (%) |
| --- | --- | --- | --- | --- |
| Unknown | 4.17 | 120.134 | 108.367 | 18.43 |
| Unknown | 6.83 | 444.836 | 179.051 | 30.46 |
| Galactosamine | 10.17 | 22.212 | 8.765 | 1.49 |
| Glucosamine | 12.25 | 28.760 | 13.650 | 2.32 |
| Glucose | 14.50 | 15.189 | 7.612 | 1.29 |
| Unknown | 46.50 | 58.080 | 19.053 | 3.24 |
| Unknown | 50.00 | 534.195 | 219.448 | 37.33 |
| Unknown | 63.33 | 16.599 | 6.657 | 1.13 |
| Unknown | 69.58 | 51.572 | 25.270 | 4.30 |

**Table S3**: High pH-chromatography data from *F. tularensis* capsule.
