# Supplementary material for: Identification, Characterization and Immunogenicity of an O-Antigen Capsular Polysaccharide of Francisella tularensis
Source: PLoS One. 2010 Jul 6;5(7):e11060. doi: 10.1371/journal.pone.0011060 (PMC2897883; doi:10.1371/journal.pone.0011060)
Supplement: Table S4 — Summary of GC-MS data for TMS derivatives of F. tularensis capsule. (0.03 MB DOC) [file pone.0011060.s014.doc]

**Table S4**: Summary of GC-MS data for TMS derivatives of *F. tularensis* capsule.

| Peak name | Ret. time (min) | Relative Area  (%) |
| --- | --- | --- |
| ^1^QuiNAc | 22.69 | 22.47 |
| ^1^QuiNAc | 23.08 | 9.07 |
| ^1^HexNAcAN | 23.73 | 8.08 |
| ^1^HexNAcAN | 23.9 | 11.02 |
| ^2^Mannitol | 26.34 | 49.36 |

^1^QuiNAc: 2-acetamido-2,6-dideoxy-D-glucose, HexNAcAN: 2-acetamido-2-deoxy-D-hexuronamide.

^2^Mannitol was included as an internal standard.
