# Supplementary material for: Identification, Characterization and Immunogenicity of an O-Antigen Capsular Polysaccharide of Francisella tularensis
Source: PLoS One. 2010 Jul 6;5(7):e11060. doi: 10.1371/journal.pone.0011060 (PMC2897883; doi:10.1371/journal.pone.0011060)
Supplement: Table S5 — Summary of GC-MS data for TMS derivatives of F. tularensis LPS. (0.03 MB DOC) [file pone.0011060.s015.doc]

| Peak name | Ret. time (min) | Relative Area  (%) |
| --- | --- | --- |
| Ribose | 13.32 | 1.04 |
| C14:0 | 16.72 | 1.32 |
| Mannose | 18.18 | 13.41 |
| Glucose | 21.61 | 29.81 |
| C16:0 | 21.8 | 5.53 |
| Glucose | 22.11 | 12 |
| ^1^Mannitol | 22.93 | 13.06 |
| ^2^Kdo | 26.33 | 4.91 |
| ^2^GlcNAc, 3-OH C16:0 | 26.58 | 6.67 |
| 3-OH C18:0 | 30.7 | 12.26 |

**Table S5**: Summary of GC-MS data for TMS derivatives of *F. tularensis* LPS.

^1^Mannitol was included as an internal standard.

^2^Kdo: 3-deoxy-D-*manno*-octulosonic acid, GlcNAc:*N*-acetyl-glucosamine
